# Uncovering the Lipid Interface in Neurotransmission: Single Molecule Measurements of Neurotransmitters Interacting with Membranes Reveal Species Dependent Membrane Binding

**DOI:** 10.1002/advs.202515727

**Published:** 2025-11-12

**Authors:** Thomas L. Derrien, Aneeth Kakkanattu Arunkumar, Rosalind Cross, Emilie Sunnucks, Frank Vollmer

**Affiliations:** ^1^ Department of Physics and Astronomy Living Systems Institute University of Exeter Exeter EX4 4QD UK

**Keywords:** membrane interactions, neurotransmitters, optical sensors, single molecule sensors, whispering gallery mode sensors

## Abstract

Neurotransmitters (NTs) have traditionally been understood to act via aqueous‐phase receptor binding, but growing evidence suggests the lipid bilayer plays an active role in modulating their signalling. Here, single‐molecule measurements are presented using whispering gallery mode (WGM) optical sensors to directly observe NT interactions with supported lipid bilayers. Aromatic NTs, such as histamine, dopamine, epinephrine, and L‐norepinephrine, exhibit stable, step‐like resonance shifts, indicating sustained membrane association, while amino acid‐like NTs generate only transient interactions. Kinetic analyses of spike and step events reveal species‐dependent association and dissociation rates. Complementary Langmuir trough experiments confirm membrane partitioning by the NT histamine, but not by glycine. These findings support a model of selective membrane partitioning in neurotransmission and highlight the bilayer as an active participant in NT signalling. This work advances the understanding of NT–membrane interactions and provides a platform for probing non‐canonical modes of synaptic communication.

## Graphical abstract

Using single‐molecule whispering gallery mode sensors, neurotransmitter‐specific membrane binding signatures are measured that reveal intrinsically distinct interaction kinetics and orientations on a lipid membrane.

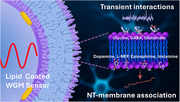

## 1 Introduction

Neurotransmitters (NTs) are essential chemical messengers responsible for synaptic communication in the nervous system. They are released from presynaptic neurons into the synaptic cleft, where they bind to receptors on the postsynaptic membrane and modulate neuronal excitability, ultimately shaping neural signalling, plasticity, and behavior.^[^ ^1^ ^]^ While the canonical model of neurotransmission focuses on receptor binding in the aqueous phase, a growing body of evidence suggests that the surrounding lipid bilayer is not merely a passive scaffold, but plays an active and selective role in shaping neurotransmitter dynamics.^[^ ^2^, ^3^, ^4^ ^]^ Several studies using surface plasmon resonance (SPR), fluorescence spectroscopy, and molecular simulations have reported direct interactions between NTs such as dopamine, serotonin, histamine, and glutamate, and model lipid membranes.^[^ ^5^, ^6^, ^7^, ^8^, ^9^, ^10^, ^11^, ^12^ ^]^ These studies point to a range of driving forces, including electrostatic attraction, hydrogen bonding, and specific headgroup interactions. For example, serotonin has been shown to associate with negatively charged membranes primarily through electrostatic interactions rather than hydrophobic partitioning,^[^ ^11^ ^]^ while dopamine shows a preference for phosphatidylserine‐rich bilayers.^[^ ^9^, ^10^ ^]^ Glutamate has also been reported to form hydrogen bonds with lipid phosphate and carbonyl groups,^[^ ^8^ ^]^ and even induce structural changes in phospholipid assemblies.^[^ ^12^ ^]^ However, these ensemble‐based approaches average over large populations of molecules and lack the temporal resolution necessary to resolve transient or heterogeneous binding events.

The possibility that membranes can transiently sequester or retain neurotransmitters introduces an additional layer of regulation in neurotransmission. Acting as a dynamic reservoir, the lipid bilayer may locally concentrate NTs at or within the membrane, thereby modulating their spatial distribution, receptor accessibility, and signalling duration. Studies of membrane‐partitioning drugs lend support to the idea that even modest physicochemical interactions with the bilayer can influence receptor function and signalling efficacy.^[^ ^13^ ^]^ If neurotransmitter accumulation at the membrane represents a general mechanism, it could significantly affect diffusion kinetics, clearance rates, and the timing of receptor engagement, factors critical for the fine‐tuning of synaptic transmission. Theoretical models have further proposed that NTs may modulate receptor behavior not only through direct binding but also by altering bilayer properties in a manner analogous to endogenous anesthetics.^[^ ^4^, ^14^ ^]^ This dual mechanism of action expands the conceptual framework of neurotransmission, encompassing both direct receptor interaction and membrane‐mediated modulation. In parallel, a wide range of psychoactive compounds, including antipsychotics^[^ ^15^ ^]^ and general anesthetic agents,^[^ ^16^, ^17^ ^]^ are known to partition into lipid bilayers and alter bilayer physical parameters such as fluidity, thickness, and receptor accessibility. Although often underappreciated, these membrane‐mediated effects may contribute substantially to drug efficacy and neurotransmitter action. Despite this, the direct experimental characterization of neurotransmitter–membrane interactions remains limited, particularly under physiologically relevant conditions.

To address this shortcoming, new methods are required that can resolve NT–membrane interactions at the single‐molecule level, correlate them with ensemble‐level membrane perturbations, and evaluate theoretical predictions. For example, experimental data using surface plasmon resonance (SPR) and neutron reflectivity (NR) measurements have shown that serotonin can penetrate lipid bilayers composed of phosphatidylcholine (PC) and phosphatidylglycerol (PG) while γ‐aminobutyric acid (GABA) does not.^[^ ^18^ ^]^ However, isothermal calorimetry experiments revealed that in the presence of PC with 10% PG, GABA does adsorb to the membrane interface.^[^ ^3^ ^]^ Furthermore, the amphiphilic neurotransmitter serotonin has been shown to interact with negatively charged membranes primarily through electrostatic, rather than hydrophobic, interactions^[^ ^11^ ^]^ and to interact preferentially with phospholipid headgroups in DPPC monolayers.^[^ ^19^ ^]^

Single‐molecule detection techniques have the potential to bridge these seemingly conflicting observations by directly capturing low‐affinity interactions that are often averaged out in ensemble measurements. By combining molecular‐level resolution with real‐time kinetic analysis, such methods could clarify the mechanistic basis of neurotransmitter–membrane association and test emerging theoretical models that include 2D diffusion, partitioning, and membrane‐mediated signalling effects. As a step toward this goal, plasmonic optical fibers have recently been used to achieve single‐molecule detection of dopamine,^[^ ^20^ ^]^ demonstrating the feasibility of non‐invasive, label‐free neurotransmitter sensing in solution, albeit in the absence of lipid bilayers. Other optical techniques, particularly whispering gallery mode (WGM) sensors, offer a powerful approach to detecting individual NT events with sub‐millisecond temporal resolution and single‐molecule sensitivity. WGM sensors detect individual molecular interactions by monitoring shifts in the optical resonance of light circulating within a microscale dielectric cavity such as a glass microsphere.^[^ ^21^, ^22^, ^23^, ^24^ ^]^ When a molecule binds at or near the microsphere's surface, perturbations cause a measurable shift in the resonant wavelength that can be tracked in real‐time with high sensitivity. Using ≈ 100 µm glass microspheres, we have used these optoplasmonic WGM sensors to detect single molecule events such as single enzyme catalysis,^[^ ^24^, ^25^, ^26^ ^]^ binding of single amino acids,^[^ ^27^ ^]^ and monitored the optical response of bacteriorhodopsin membranes.^[^ ^28^ ^]^ Recently, we employed plasmonically enhanced WGM sensors to detect NTs in free solution.^[^ ^29^, ^30^ ^]^ Here, we extend this platform to directly observe NT–lipid membrane interactions. The integration of lipid bilayers onto WGM sensors offers a new capability, providing a powerful platform to test key theoretical predictions arising from the membrane‐dependent transmission model. Such as, for example, the prediction that neurotransmitters may undergo a dimensionality shift upon approaching the membrane, transitioning from 3D diffusion in solution to 2D lateral diffusion along the membrane surface.^[^ ^2^, ^31^, ^32^ ^]^ This shift is expected to enhance receptor binding rates by increasing the local NT concentration at the membrane interface, potentially reshaping our understanding of synaptic signalling.

In this study, we use single‐molecule WGM sensing to systematically investigate the membrane affinity of neurotransmitters with distinct chemical characteristics. Our results reveal that aromatic NTs, including histamine, epinephrine, and L‐norepinephrine, exhibit sustained interactions with lipid membranes, while amino acid‐like NTs, such as glycine, GABA, and glutamate, only interact transiently and do not measurably perturb membrane structure. Langmuir trough measurements further support this distinction, showing a significant surface pressure increase following aromatic NT injection, indicative of lipid partitioning or adsorption. In contrast, control and glycine injections result in minimal or transient effects, confirming the specificity of membrane engagement by an aromatic NT. While previous ensemble studies have reported membrane interactions of neurotransmitters, our single‐molecule measurements uniquely reveal both transient and sustained binding dynamics with lipid bilayers. To our knowledge, this is the first demonstration of neurotransmitter–membrane interactions resolved at the single‐molecule level at physiologically relevant concentrations down to nM to fM range, revealing dynamic behaviors not accessible with ensemble techniques.

These findings provide strong experimental support for a membrane‐partitioning model of neurotransmission and suggest that membrane association may be a selective feature that distinguishes classes of NTs based on their physicochemical properties and receptor types. Our data also imply that lipid composition and membrane mechanics could play underappreciated roles in shaping synaptic signalling and receptor access. This integrated methodology, combining single‐molecule and ensemble‐scale techniques, establishes a basis for re‐evaluating neurotransmission through the lens of NT–membrane interactions.

## 2 Results

To investigate the range and nature of neurotransmitter (NT) interactions with lipid membranes, we tested seven NTs using supported lipid bilayers (SLBs) composed of EggPC and cholesterol in a 9:1 molar ratio. These NTs can be broadly grouped into two categories: amino acid‐like or polar types (GABA, glycine, and glutamate) and aromatic or amphipathic types (epinephrine, L‐norepinephrine, histamine, and dopamine). Interactions were probed using optoplasmonic WGM sensors, which consisted of 85‐90 µm diameter glass microspheres functionalised with plasmonic gold nanorods to achieve single‐molecule sensitivity.

**Figure 1:**
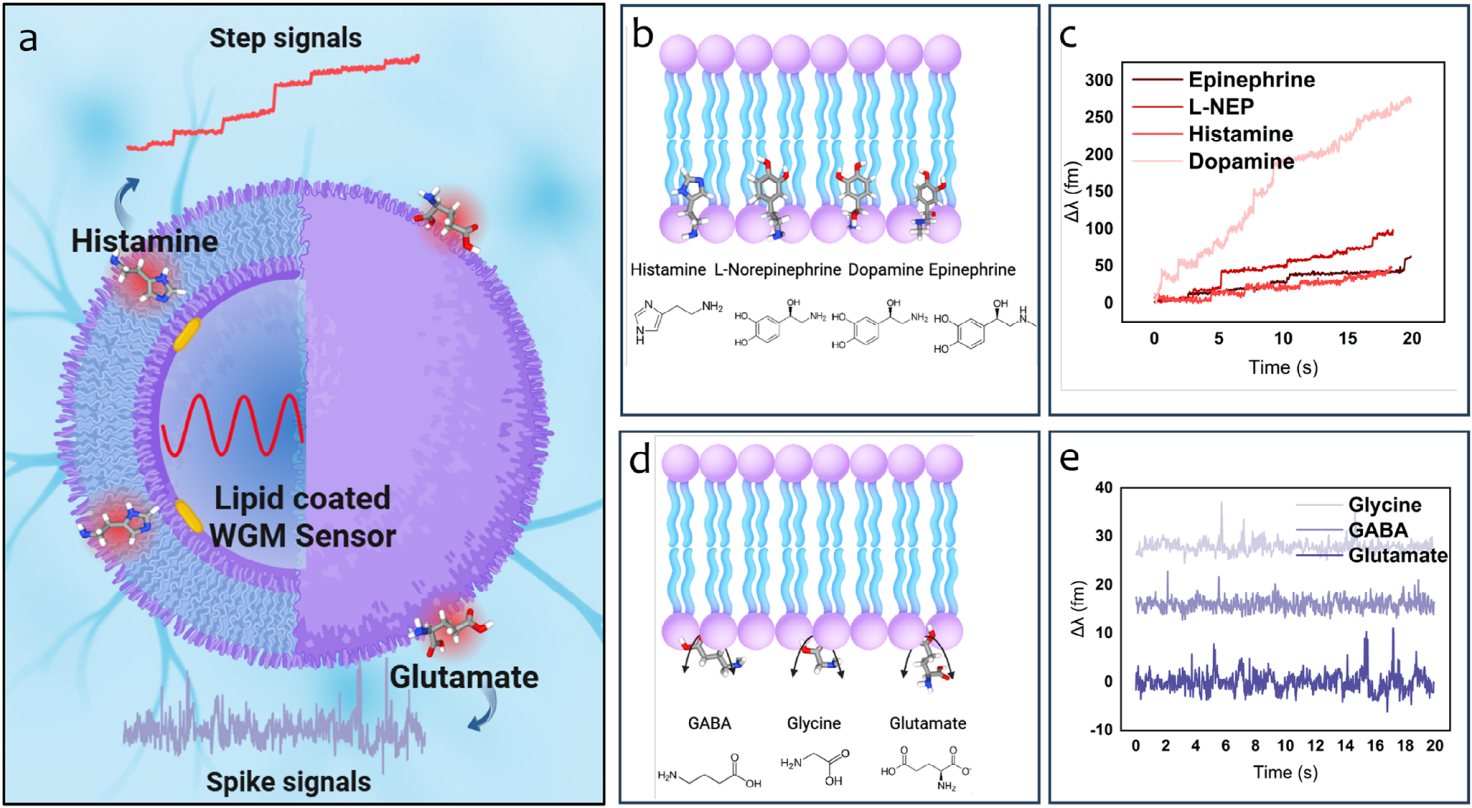
Sensing scheme depicting NTs interacting with a SLB on the WGM sensor with membrane partitioning NT (histamine), and non‐partitioning NT (glutamate) and their corresponding traces (a). cMembrane partitioning NTs (b) and sample WGM traces showing step‐like signals (c). Non partitioning NTs (d) and sample WGM traces, showing spike‐like signals (e).

**Figure 2:**
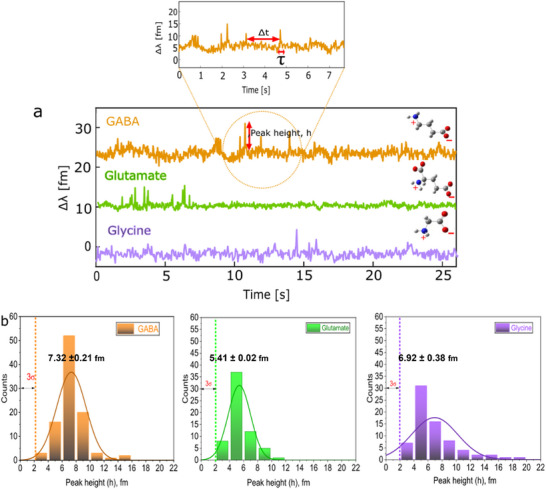
Interaction GABA, glutamate, and glycine with lipid bilayer. Spike signals obtained upon addition of NTs to PBS (a). Resonance shift (Δλ) distribution of the amplitude of spike signals of GABA (orange), glutamate (green), and glycine (purple). All histograms are fit with a Gaussian distribution.

The SLB was formed by incubating the plasmonic resonator in a solution of liposomes (Figure S1, Supporting Information) suspended in phosphate‐buffered saline (PBS), resulting in membrane deposition on the microsphere surface (**Figure** **1**a). The successful formation of the SLB was monitored by tracking shifts in the WGM resonance position. A total shift of ≈82 pm was observed, corresponding to a calculated increase in resonator diameter of ≈4 nm, consistent with the expected thickness of a lipid bilayer and indicating near‐complete coverage (Figure S2, Supporting Information). WGM resonance shifts occur when individual molecules enter the optical near field of the plasmonic nanorods. Transient interactions, where a molecule briefly enters this region before diffusing away, produce spike‐like signals (Figure 1C), whereas stable interactions, where the molecule remains within the near field, generate step‐like shifts (Figure 1E). In the presence of a lipid bilayer, this signal distinction enables us to differentiate NTs that remain associated with the membrane from those that dissociate rapidly. Experiments with the amino acid‐like NTs—GABA, glycine, and glutamate—revealed exclusively transient interactions in the presence of the EggPC:cholesterol SLB. This is illustrated in representative signal traces shown in **Figure** **2**a. The strength and nature of these interactions can be inferred from both the amplitude and duration (dwell time, τ) of the spike signals. When comparing the histograms of signal heights at 10 nM, no significant differences were observed between the three NTs (Figure 2b). Since signal amplitude is primarily determined by molecular polarizability and proximity to the plasmonic near field, and given that the polarizabilities of these NTs are broadly similar, we infer that they interact at comparable distances from the nanoparticle surface. This suggests that the lipid bilayer restricts the NTs to surface‐level interactions, preventing direct contact with the nanorod. This interpretation is supported by control experiments performed in the absence of the lipid bilayer. Under identical conditions, these NTs generated stable, step‐like signals indicative of direct and prolonged binding to the nanorod surface (Figure S5, Supporting Information). The shift from step‐like to spike‐like signals in the presence of the membrane confirms the modulatory role of the lipid bilayer in governing NT interaction dynamics. To quantify these interactions, we conducted a survivor function analysis of key kinetic parameters, including the inter‐spike interval (Δt) and the dwell time (τ). This analysis provides estimates of NT–membrane interaction rates and yields insight into the temporal characteristics of binding events. These measurements are particularly relevant for understanding the kinetics of NT diffusion and receptor engagement at synaptic membranes, where both transient and sustained interactions may influence signal propagation and specificity.

**Figure 3:**
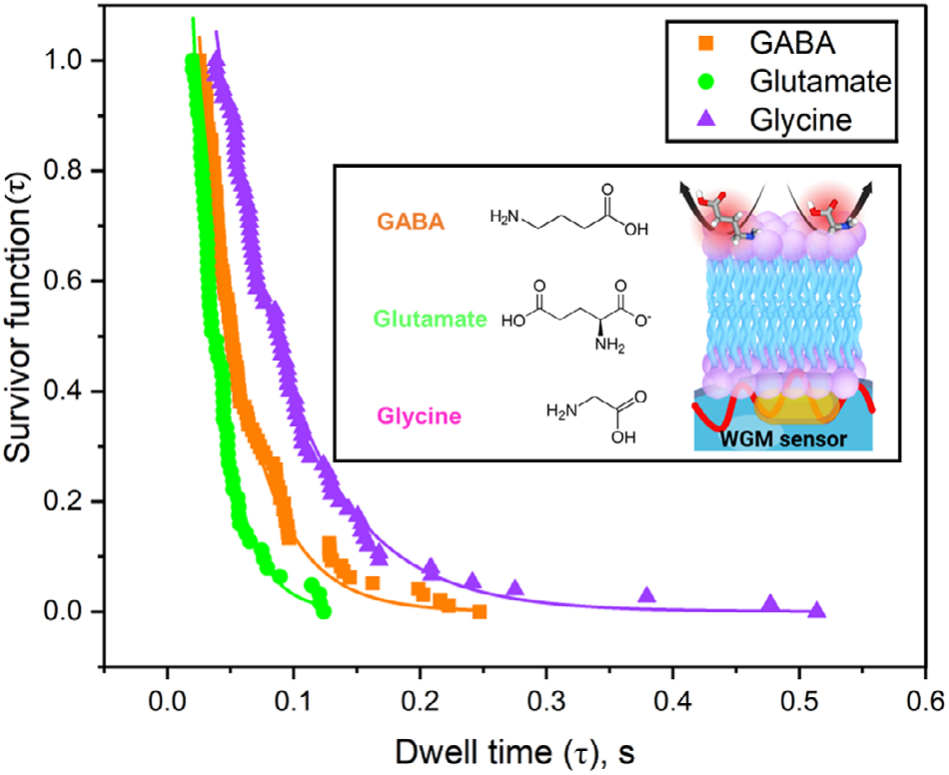
Survivor plot of dwell time statistics obtained at 10 nM. WGM resonance shift statistics of GABA, glutamate, and glycine. Chemcial structures and schematic illustration of NT interaction (Inset).

Kinetic analysis was conducted by evaluating the survivor function of the interaction dwell times, τ (Figure 2A). The survivor function, *S*(τ), defined as *P*(*T* > τ), where T is a continuous random variable, describes the probability that a molecule remains associated with the membrane beyond a given time interval. The exponential fitting of *S*(τ) was used to calculate mean dwell times for GABA, glycine, and glutamate (**Figure** **3**). Among these, glycine exhibited the longest average dwell time, while glutamate had the shortest. A longer dwell time typically indicates stronger or more persistent interaction with the membrane surface. Given that the neurotransmitters studied are zwitterionic and that the membrane model used (EggPC:cholesterol), presents a neutral or slightly negative surface potential, these interactions are likely dominated by electrostatic forces rather than hydrophobic partitioning. All three neurotransmitters possess strongly negative LogP values, indicating low lipophilicity and an inability to penetrate deeply into the hydrophobic membrane core. Thus, they are expected to remain confined to the aqueous phase or interact only at the membrane interface. Interestingly, although glutamate has the most negative LogP value and is therefore the least lipophilic NT in this group, it displays the shortest dwell time. Glycine, with a higher LogP than GABA, showed the longest dwell time, suggesting that LogP alone does not fully explain membrane interaction strength for polar NTs. These findings imply that factors beyond lipophilicity, such as molecular size, flexibility, charge distribution, or hydrogen‐bonding capability, may play a significant role in governing weak surface‐level interactions. It is also important to recognize that membrane interactions occur at the hydrophilic headgroup region of the bilayer, where electrostatic forces and specific polar interactions are more relevant than hydrophobic partitioning.

A single exponential fit of the form *y* = *Ae* ^−*kt*^ to the dissociation data suggests that non‐membrane‐binding neurotransmitters interact with lipid bilayers through a simple one‐step dissociation process. Full details of the kinetic fitting procedures and parameters derived from survival analyses are provided in the Supplementary Information (Tables S1–S4, Supporting Information). The off‐rates determined for GABA, glutamate, and glycine were 27.0 s^−1^ (0.037 s), 44.5 s^−1^ (0.0225 s), and 16.1 s^−1^ (0.0622 s), respectively. Taking the inverse of these rates gives the mean interaction times, showing that glutamate has the shortest interaction time, followed by GABA, while glycine has the longest interaction time of the three. In addition to their low affinity for membranes, electrostatic interactions also play a role in how these neurotransmitters associate with lipid bilayers. As previously noted, the lipid bilayer carries a net negative surface charge at physiological pH (7.3) due to its headgroup composition. Glutamate has two negatively charged carboxylate groups at this pH, which leads to strong electrostatic repulsion from the membrane and contributes to its fast dissociation and short interaction time. This is consistent with its highly negative logP value of –3.69, indicating strong hydrophilicity. GABA, which has a logP of –3.00 and only one carboxylate group, experiences less repulsion and remains associated with the membrane slightly longer. Glycine, despite being the most hydrophilic of the three (logP –3.21), shows the longest interaction time. This inversion of the expected trend between logP and dwell time suggests that factors beyond bulk hydrophobicity, such as molecular size, conformational flexibility, and specific local interactions, may play a significant role. Glycine's small molecular size may facilitate a closer approach and transient stabilization at the membrane interface despite its high hydrophilicity.

Further survival analysis was undertaken to determine the “on‐rates” of these interactions. The survivor function analysis of the inter‐spike interval, Δ*t*, describes the probability that a binding event has not occurred within a given time interval. These distributions were fitted with exponential models to extract on‐rate constants, which reflect the frequency of neurotransmitter‐induced interactions. Survivor plots for Δ*t*, corresponding to GABA, glutamate, and glycine are presented in Figure S4 (Supporting Information). Each neurotransmitter was tested at three concentrations: 10 nM (grey squares), 1 μM (red circles), and 100 μM (blue triangles). Data for each concentration were fitted with color‐coded exponential models. Initial attempts using single‐phase exponential fits did not adequately capture the observed dynamics. Improved fits were obtained using a bi‐exponential model of the form

(1) $$y=Ae^{-k_{1}t}+Be^{-k_{2}t}$$

where *k*_1_ and *k*_2_ are two distinct on‐rate constants. The need for a two‐phase model suggests that neurotransmitter interactions with the membrane are not governed by a simple, one‐step mechanism but instead involve at least two kinetically distinct processes. This contrasts with the monoexponential character of the dissociation phase, reflecting a simpler, unified unbinding mechanism and highlighting the asymmetry and complexity of NT–membrane interactions.

This bi‐exponential behavior also contrasts with previously reported single‐phase kinetics for the interaction of GABA, glycine, and glutamate with CTAB‐coated gold nanorods.^[^ ^29^, ^30^ ^]^ In the current study, the two extracted on‐rate constants, summarized in **Table** **1**, indicate the presence of two modes of interaction: a rapid phase, likely corresponding to transient surface association, and a slower phase that may reflect molecular rearrangement or transient lateral diffusion across the membrane. Across all concentrations and neurotransmitters tested, *k*_1_ was consistently greater than *k*_2_, typically differing by at least an order of magnitude.

**Table 1:** On‐rates extracted from the survivor function plots.

Neurotransmitters	Concentration	On‐rate (k_1_) [s^−1^]	On‐rate (k_2_) [s^−1^]
GABA	10 nM	0.47 ± 0.02	0.03 ± 0.008
	1 µM	0.57 ± 0.06	0.08 ± 0.008
	100 µM	0.55 ± 0.05	0.05 ± 0.002
Glutamate	10 nM	1.17 ± 0.07	0.02 ± 0.002
	1 µM	0.21 ± 0.03	0.01 ± 0.004
	100 µM	0.87 ± 0.09	0.03 ± 0.003
Glycine	10 nM	4.14 ± 0.4	0.04 ± 0.001
	1 µM	1.4 ± 0.1	0.06 ± 0.001
	100 µM	0.34 ± 0.02	0.45 ± 0.002

Importantly, the observed trends cannot be rationalized by logP alone because most neurotransmitters are polyprotic, and their charge state depends strongly on pH. We therefore interpret the concentration dependence and step/spike behavior using the distribution coefficient at the experimental pH (logD_pH_), together with pK_*a*_‐based speciation, charge distribution, and hydrogen‐bonding capacity. For amino acid–like NTs (GABA, Glu, Gly; pH 7.4), the dominant zwitterionic or anionic forms exhibit high hydration penalties and strong water H‐bonding, giving very low logD_7.4_ and predominantly transient, spike‐type interactions. In contrast, aromatic amines (dopamine, epinephrine, norepinephrine; pH 6.5) remain cationic with hydrophobic aromatic cores, supporting interfacial residence and step‐type events. Histamine illustrates why logP is misleading: at pH 6.5, a significant fraction is dicationic, increasing hydration and electrostatic repulsion from the headgroup dipole, thus reducing interfacial binding despite a favorable neutral‐species logP. Specific interfacial interactions further bias outcomes: cation–phosphate attraction, H‐bonding to lipid headgroups, and CH–π/dispersion contacts stabilize shallow, oriented states, whereas β‐hydroxyl groups in epinephrine/norepinephrine increase polarity, reducing partitioning relative to dopamine.

Overall, considering pH‐dependent speciation, molecular electrostatics, and interfacial interactions can account for the apparent discrepancies between logP and experimental kinetics while remaining consistent with TE/TM anisotropy, which suggests interfacial, rather than deeply intercalated, orientations.

This demonstrates the capacity of optoplasmonic WGM sensors to resolve complex kinetic behaviors at the single‐molecule level. Their high sensitivity permits the detection of subtle diffusion processes along the membrane surface, which may be especially relevant for weakly associating neurotransmitters. Future studies incorporating membrane‐embedded receptors may enable direct visualization of receptor‐mediated events, offering deeper insight into the molecular dynamics of synaptic transmission.

Together, these results highlight the importance of considering both electrostatic and kinetic parameters when assessing NT–membrane interactions. While LogP provides a useful approximation of lipophilicity, it does not adequately account for the complexities of molecular behavior at the bilayer interface.

**Figure 4:**
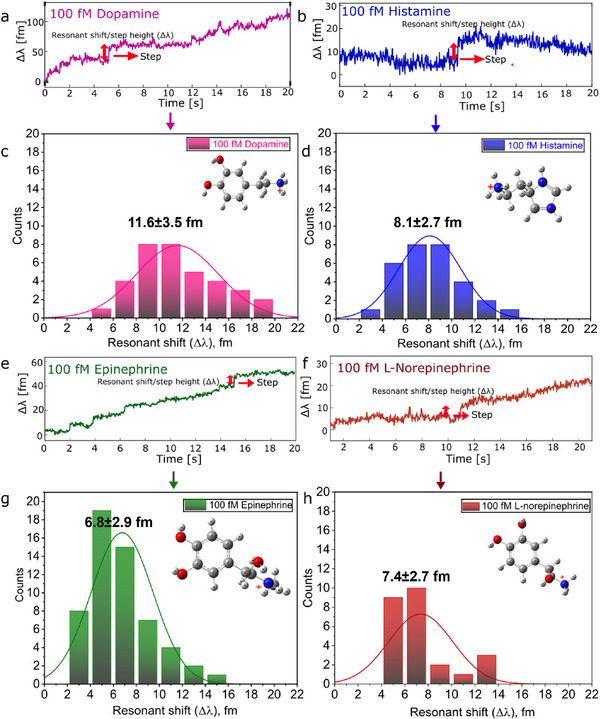
High‐affinity step signals obtained when a) dopamine, b) histamine, e) epinephrine, and f) L‐norepinephrine interact with supported lipid bilayers. The concentration of each neurotransmitter was 100 fM. Corresponding distributions of resonant wavelength shifts are shown for c) dopamine, d) histamine, g) epinephrine, and h) L‐norepinephrine. Each histogram was fitted with a normal distribution.

To investigate membrane partitioning behavior, experiments were conducted with aromatic neurotransmitters (NTs) at concentrations of 100 fM, 100 pM, and 10 nM (**Figure** **4**; Figure S6, Supporting Information). Unlike amino acid‐like NTs, compounds such as epinephrine, norepinephrine, dopamine, and histamine produced clear step‐like resonance shifts, indicative of sustained interactions with the lipid bilayer. Control experiments were conducted to confirm that the observed signals were not attributable to direct nanorod binding (Figure S5, Supporting Information).^[^ ^29^, ^30^ ^]^ Such signals, absent in prior experiments with GABA, glycine, or glutamate, suggest stable membrane association. The persistence of these shifts points to long‐lived single NT binding events, likely at or near the lipid headgroups, as supported by previous studies using Langmuir monolayer and SPR techniques.^[^ ^6^, ^11^, ^19^ ^]^ The amphipathic nature of these aromatic NTs, particularly the presence of benzene or imidazole rings, promotes partitioning into the bilayer. These interactions are further stabilized by hydrogen bonding between the NT headgroups and the polar components of the lipid interface. Additionally, the steric bulk of the aromatic ring may enhance binding by promoting close packing within the membrane and potentially altering local bilayer structure. These findings align with predictions from molecular dynamics simulations and LogP‐based lipophilicity estimates. Previous umbrella sampling simulations have demonstrated that aromatic NTs such as dopamine and serotonin have favorable free energy profiles for insertion into phospholipid bilayers, consistent with experimental evidence of partitioning and strong membrane affinity.^[^ ^2^ ^]^

Representative traces and corresponding histograms of WGM step heights across concentrations are shown in Figure 4. No significant difference in step amplitude was observed with changing NT concentration (Figure SI). Interestingly, the step heights recorded in the presence of lipid membranes were greater than those reported for the same NTs binding directly to plasmonic nanorods in the absence of a bilayer.^[^ ^30^ ^]^ This observation is counterintuitive, as the refractive index contrast between lipid and NT is lower than that between water and NT, and thus a smaller WGM shift would typically be expected within the bilayer environment. We hypothesize that the observed increase in WGM shift may not be due solely to the presence of the NT near the nanorod but also to local bilayer compression. The association of the NT with the membrane may restrict lipid motion, increasing local density and inducing a measurable shift in resonance. This interpretation is supported by prior studies on serotonin, which reported a decrease in lipid area per molecule upon NT association with membranes.^[^ ^18^ ^]^ To further investigate this hypothesis, Langmuir trough experiments were carried out to measure surface pressure changes in DOPC monolayers upon exposure to both aromatic and amino acid‐like NTs. Constant‐area conditions were maintained to ensure that pressure changes could be attributed to molecular partitioning rather than mechanical compression. Injection of PBS alone resulted in a reproducible, sharp pressure drop due to subphase disturbance, but no subsequent increase, confirming that mechanical effects did not account for pressure rises observed in NT trials. Following histamine injection, a sustained and reproducible increase in surface pressure was observed, amounting to ≈1.07 mN m^−1^ after correction for the initial injection artefact. This result is consistent with insertion of histamine into the monolayer, altering lateral packing and increasing compressive forces within the interface. The aromatic imidazole ring and positive charge at physiological pH likely mediate favorable interactions with both the negatively charged headgroup region and the hydrophobic interface of the lipid molecules.

In contrast, glycine injection produced only a minimal increase in surface pressure (0.07 mN m^−1^), which was gradual in nature and lacked a distinct kinetic signature. This behavior is consistent with a lack of appreciable interaction between glycine and the lipid film, suggesting that any observed pressure variation may stem from weak, non‐specific interactions or baseline drift rather than membrane association.

In addition to dwell time analysis, survivor functions were used to evaluate the frequency of step‐like events, providing a measure of the step‐associated on‐rate. Because step signals reflect long‐lived membrane residency rather than brief transient contacts, the dissociation (off) rate is not readily resolved from the WGM signal and is therefore not meaningful in this context. Instead, we analyzed the distribution of time intervals between successive step events (Δt), fitting the resulting survivor curves to single‐ or double‐exponential decay models (Figure S9, Supporting Information). Most neurotransmitters exhibited bi‐exponential on‐rate kinetics for step signals, consistent with a two‐phase association process likely reflecting an initial 3D diffusion step from solution, followed by lateral 2D diffusion along the membrane surface. This bi‐exponential behavior is a hallmark of dimensionality shift, in which molecules transition between diffusive regimes with distinct spatial constraints and timescales. Notably, L‐NEP deviated from this trend, exhibiting a clear single‐exponential Δ*t* distribution. This suggests that although L‐NEP forms stable membrane associations, these occur through a single dominant mode, potentially lacking the extended 2D diffusion phase observed for other aromatic NTs. This distinction reinforces the diversity of membrane interaction modes between closely related neurotransmitters; epinephrine and L‐norepinephrine differ by only a terminal methyl group, yet this structural variation influences their membrane dynamics and may contribute to their distinct physiological effects.

Taken together, these results indicate that histamine, an aromatic, protonated neurotransmitter, exhibits a robust and specific affinity for phospholipid monolayers, while glycine, a small zwitterionic amino acid, shows negligible or indirect interaction. This distinction aligns with a broader hypothesis that neurotransmitter–membrane interactions are chemically selective and may contribute to functional roles at the synapse, particularly for neurotransmitters capable of modulating membrane structure or surface potential. These findings support the emerging view that neurotransmitters can be categorized not only by their receptor targets but also by their biophysical membrane affinities, offering potential insights into both canonical and non‐canonical modes of signalling.

To evaluate the spatial characteristics of neurotransmitter interactions with lipid membranes, we monitored three complementary optical metrics derived from bulk WGM sensing (no Au nanorod). The transverse electric (TE) and transverse magnetic (TM) shifts reflect time‐resolved changes in resonance wavelength for modes sensitive to different orientations of the evanescent field. The difference between these shifts, ΔΔλ(t), provides a measure of evolving polarization contrast, which may indicate depth‐associated interactions or molecular orientation changes.^[^ ^28^, ^33^ ^]^ The ratio R(t), defined as the TM shift divided by the TE shift, serves as an alternative indicator of anisotropy, with values greater than one suggesting enhanced vertical orientation in the membrane and more pronounced coupling to the TM mode over the TE mode. The results are shown in Figure S10 (Supporting Information). In the presence of supported lipid bilayers, histamine produced steadily rising shifts in both modes, with TM consistently exceeding TE. The difference between modes, ΔΔλ(t), increased smoothly over time, indicating the development of vertical refractive index contrast near the sensor surface. This effect is further supported by a relatively stable R(t) value greater than one, suggesting that histamine induces a depth‐associated interaction consistent with partial membrane insertion or vertical reorganisation within the bilayer. The pronounced TM‐dominant shifts observed for histamine, particularly in the presence of supported lipid bilayers, suggest a preferential vertical alignment of the molecule at the membrane interface. In our WGM sensing geometry, such polarization contrast reflects an anisotropic refractive index change that couples more strongly to the TM mode, whose electric field projects normal to the surface. To account for refractive changes due to local refractive index changes in the TE/TM anisotropy, we compare our measurements to mode‐resolved WGM responses reported for isotropic refractive‐index (RI) perturbations, which yield a near‐constant, mode‐set TE/TM shift ratio.^[^ ^34^ ^]^ By contrast, our membrane data show analyte‐dependent deviations from this isotropic baseline: histamine + membrane exhibits a markedly larger TE/TM ratio than expected for an RI‐only effect, whereas glycine + membrane remains close to the isotropic limit. This behavior is consistent with a model in which histamine adopts a partially inserted orientation, penetrating or perturbing the bilayer in a direction perpendicular to the membrane plane. In the polarisation ratio trace, histamine exhibits a distinct transient feature between 50 and 150 s, marked by an early increase in the TM/TE shift ratio followed by a gradual decline. This dynamic suggests a multiphasic interaction process. Initially, the rise in ratio implies a preferential enhancement of TM mode shifts, consistent with vertically oriented histamine molecules engaging with the membrane. As the interaction progresses, the subsequent decline in the ratio may reflect a reorientation of histamine within the membrane, lateral redistribution along the bilayer surface, or saturation of deeper binding sites. Such temporal evolution points to a complex, reorganizing association process rather than a static membrane binding event. In contrast, glycine in the same lipid‐rich condition showed closely overlapping TE and TM shifts, minimal ΔΔλ(t), and a ratio near unity throughout. These patterns suggest that glycine remains confined to the interfacial region and does not disrupt or penetrate the bilayer. While this observation does not alone confirm insertion, the sustained elevation of ΔΔλ(t) and R(t), coupled with increased signal variability and broader TE‐mode histograms, supports the hypothesis that histamine associates with the membrane in a depth‐sensitive and structurally disruptive manner. Such orientation‐specific optical contrast provides a non‐invasive proxy for assessing the membrane association mode of small molecules.

**Figure 5:**
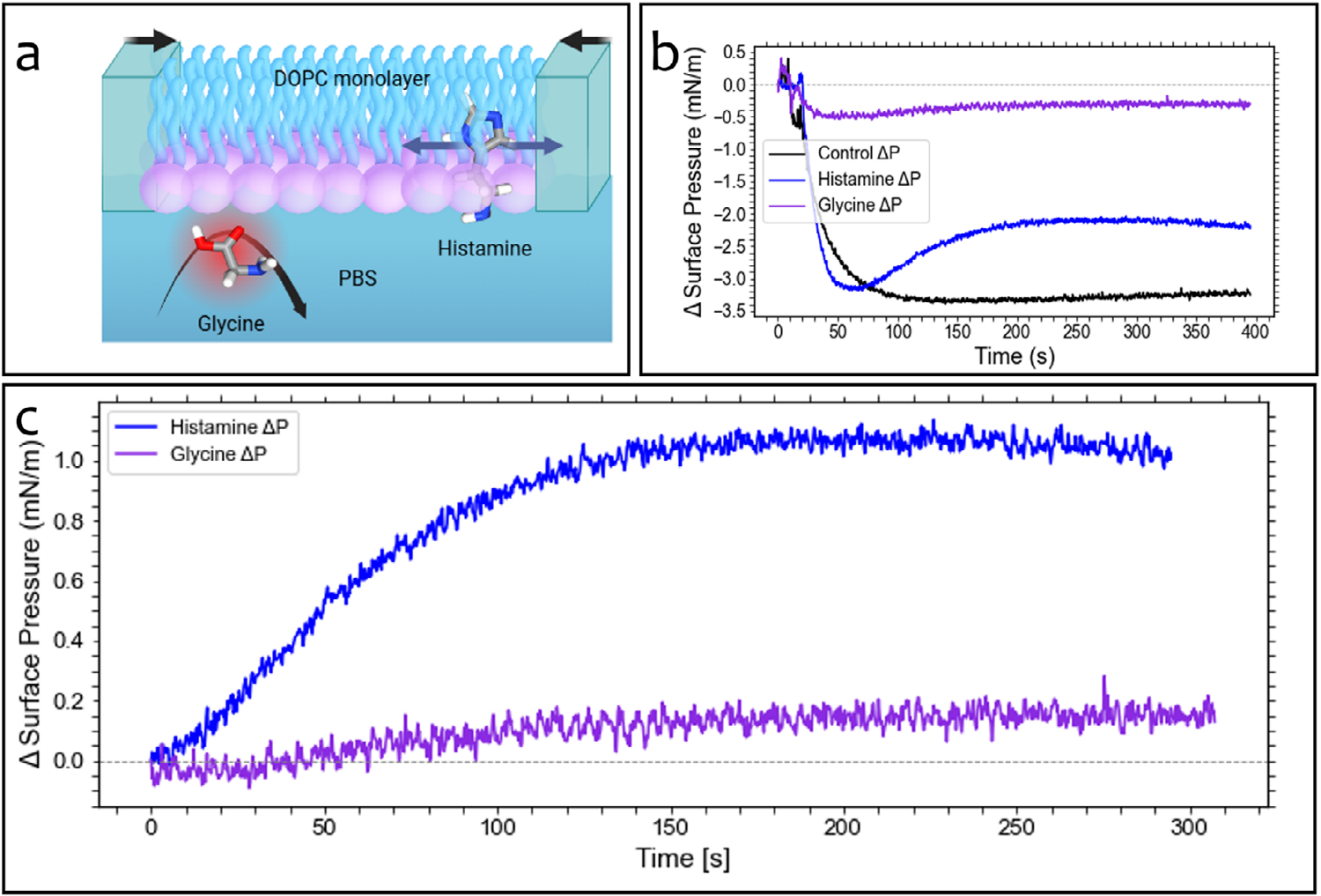
Langmuir monolayer measurements reveal differential membrane interactions between histamine and glycine. Schematic of the experimental setup: a DOPC lipid monolayer is compressed at the air–water interface while neurotransmitters (NTs) in the subphase interact with the DOPC monolayer. Aromatic NTs like histamine partition into the monolayer, while small amino acid‐like NTs such as glycine show minimal association. over time following NT injection into the subphase (a). Time traces of surface pressure (ΔΠ) following NT injections. Histamine (blue) induces a substantial and sustained increase in surface pressure, consistent with membrane insertion. Glycine (purple) produces minimal change, comparable to the control (black) (b). Zoomed view of surface pressure changes. Histamine exhibits a clear time‐dependent adsorption profile, while glycine produces a negligible response, indicating weak or absent membrane binding under these conditions (c).

**Figure 6:**
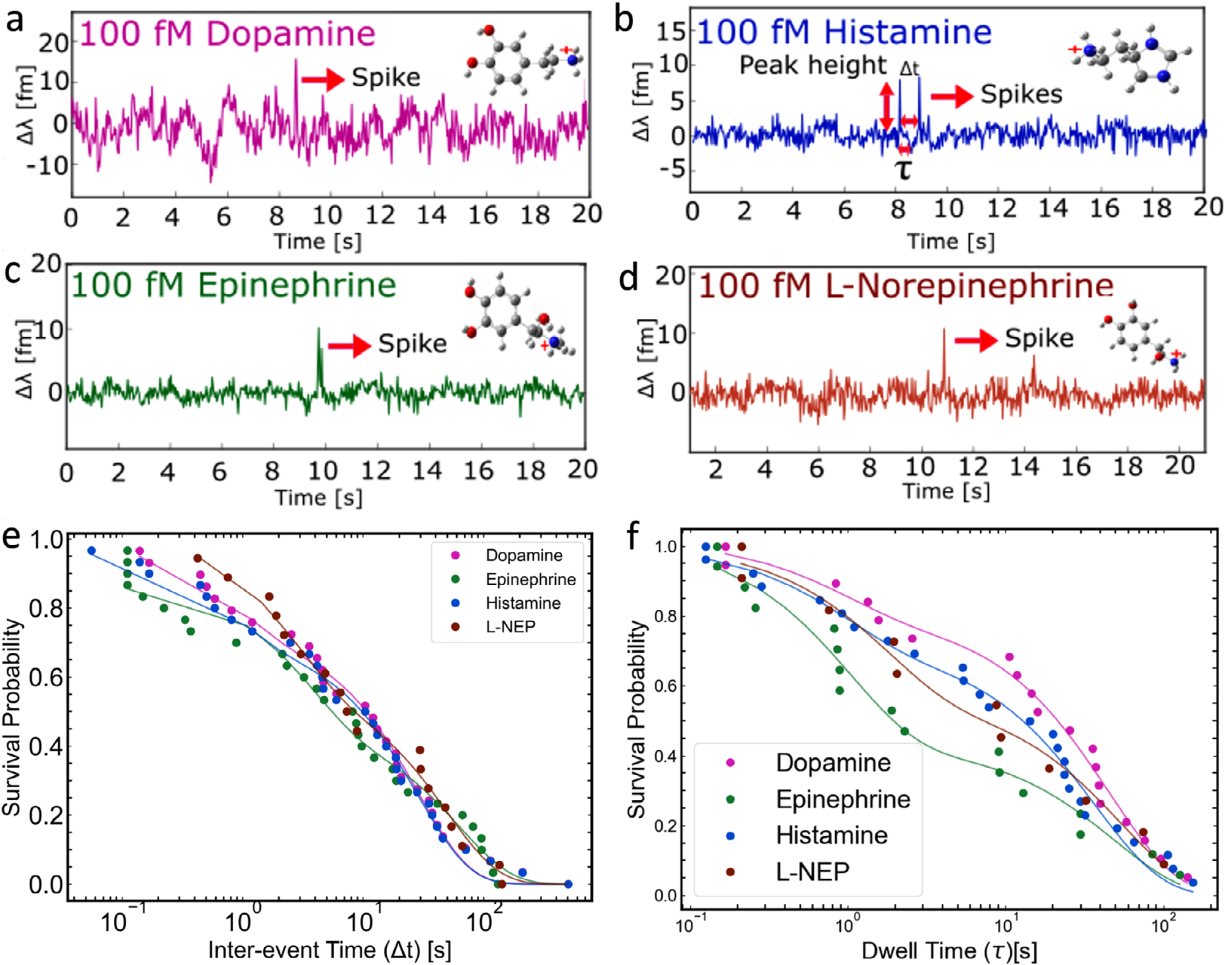
Spike signals recorded from membrane binding neurotransmitters. Panels show representative signals for: a) dopamine, b) histamine, c) epinephrine, and d) L‐norepinephrine. Survivor function for e) inter‐event time (Δt) and b) dwell time (τ) for membrane binding neurotransmitters histamine (blue), L‐norepinephrine (dark red), dopamine (magenta), and epinephrine (green), and their corresponding single exponential fit. The concentration of each neurotransmitter is 100 fM. Each experiment was performed using an individual sensor assembly comprising a SLB coated microsphere–AuNR (gold nanorod) construct. The concentration of each neurotransmitter was 100 fM.

While stable, step‐like resonance signals were the predominant feature of the WGM experimental data for aromatic neurotransmitters (NTs), spike‐like signals were also consistently observed. These spikes, recorded during measurements at 100 fM NT concentrations, represent transient interactions with the lipid bilayer that do not culminate in full membrane partitioning. **Figure** **5** and **Figure** **6** present both signal types for dopamine (pink), histamine (blue), epinephrine (green), and L‐norepinephrine (brown), highlighting the diversity in interaction modes within this group.

The coexistence of step and spike signals suggests that molecular orientation may play an important role in membrane binding. For example, protonated amine groups on NTs may experience electrostatic repulsion from the positively charged lipid headgroups, particularly at pH 6.5, the pH at which the experiment was performed to avoid polymerisation of dopamine. If an NT approaches the membrane in an unfavourable orientation, such repulsion may prevent insertion, resulting in a spike. In contrast, a favourable alignment may minimize repulsion, allowing the molecule to insert into the membrane. This model is supported by molecular dynamics simulations demonstrating the influence of orientation on membrane interaction.^[^ ^2^ ^]^

For the survivor function plots based on time separation of spike events (Δ*t*) (Figure 6e), all neurotransmitter datasets were best described by a single‐exponential decay of the form $y=Ae^{-k_{on}\Delta t}$, indicating a first‐order kinetic process. Although minor deviations were observed due to limited spike event counts, the extracted on‐rate values provide meaningful insight into the frequency of membrane‐binding interactions. The results suggest a single‐step association mechanism for membrane‐binding NTs.

The presence of single‐exponential decay for inter‐event separation (on rate) further supports the interpretation that membrane‐binding NTs undergo 3D diffusion prior to interacting with the bilayer. However, lateral diffusion on the membrane surface appears to be limited, possibly due to crowding by membrane‐bound NTs. This inhibition of further diffusion or interaction could explain the suppression of additional binding events and underscores a key mechanistic distinction from membrane‐inert NTs.

The survivor functions for the dwell time (τ) (Figure 6e) of membrane‐binding NTs were similarly best described by a single‐exponential decay of the form $y=Ae^{-k_{off}\tau }$, consistent with previous observations for non‐membrane‐binding NTs. These fits describe the probability that an NT remains associated with the membrane beyond a given time point, and the decay constants reflect the off‐rate for each neurotransmitter. The extracted values offer a quantitative measure of the dissociation kinetics.

**Figure 7:**
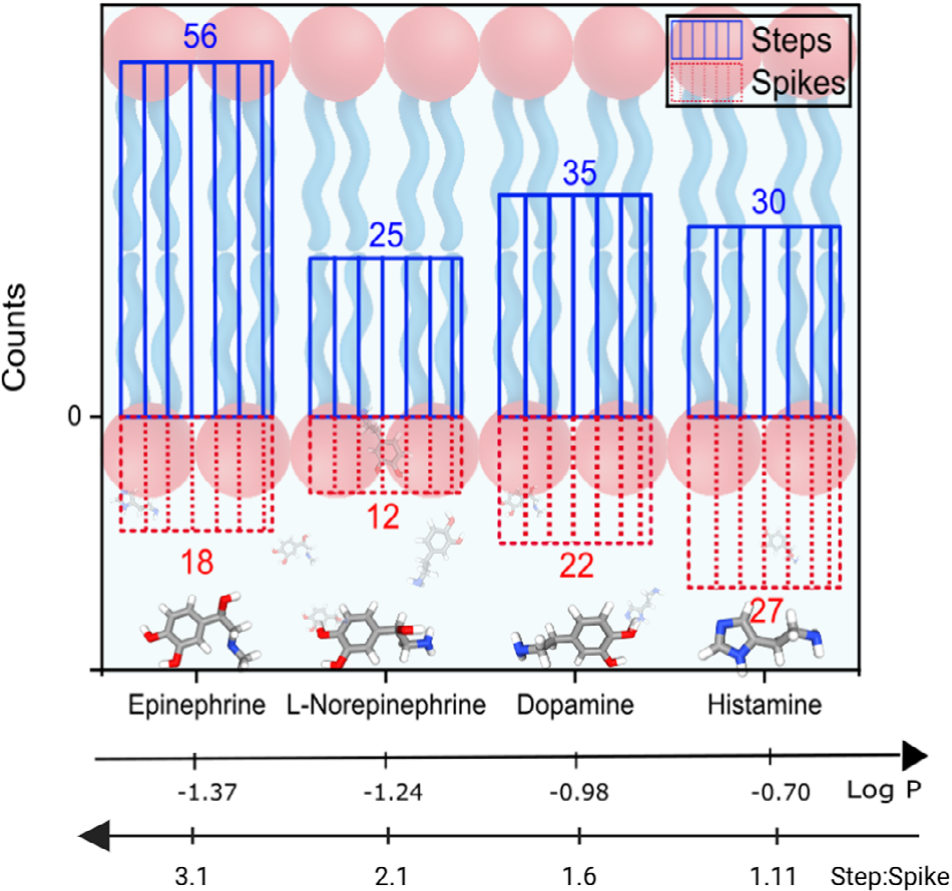
Number of step and spike signals recorded from membrane‐binding neurotransmitters at a concentration of 100 fM. A greater number of step signals compared to spike signals indicates that these neurotransmitters predominantly associate with the membrane.

Despite the detection of spike signals, the total number of step signals exceeded that of spikes for all NTs tested, suggesting that stable membrane association is the dominant mode of interaction (**Figure** **7**). For instance, dopamine and histamine yielded 35 and 30 step signals, respectively, whereas L‐norepinephrine showed the fewest. Notably, histamine displayed nearly equal numbers of step (30) and spike (27) signals. This may be explained by its structural features: compared to the other NTs, which contain a six‐carbon aromatic ring, histamine possesses a smaller imidazole ring composed of three carbon and two nitrogen atoms. This relatively less bulky structure may reduce its membrane‐binding affinity. Additionally, the presence of protonated monoamines, which contribute to spike signals through electrostatic repulsion from the protonated lipid headgroups, may account for the comparable number of spike and step signals observed for histamine.

It is useful to consider the step‐to‐spike ratio for each NT, as a higher ratio suggests stronger membrane interaction. Based on the data shown in Figure 7 at 100 fM concentration, epinephrine exhibited the highest step‐to‐spike ratio, suggesting it has the strongest membrane affinity. LogP values are commonly used to predict lipophilicity, and although histamine has the highest LogP among the tested NTs,^[^ ^35^ ^]^ it displayed a lower step‐to‐spike ratio than epinephrine, indicating that additional factors beyond lipophilicity influence membrane interaction. Counter to expectations based on LogP, we find that neurotransmitters with higher LogP values produce fewer stable step events relative to transient spikes, indicating that lower‐lipophilicity compounds may interact more robustly or persistently with the membrane interface under our experimental conditions. This inverse relationship between LogP and the step to spike ratio further supports our conclusion that bulk lipophilicity is a poor predictor of membrane interaction dynamics in this context; compounds with higher LogP values, expected to associate more strongly with lipid bilayers, in fact exhibit fewer stable (step) interactions than their more hydrophilic counterparts.

Although step signals are more numerous, spike signals provide valuable kinetic information. On‐rate and off‐rate constants were derived from survivor function analyses of spike time separation (Δ*t*) and dwell time (τ), respectively, as shown in Figure 6. These parameters offer insights into the frequency and duration of membrane engagement. Survivor function plots can further distinguish whether neurotransmitter–membrane interactions follow single or multiple kinetic processes.

The kinetic behavior of membrane‐binding neurotransmitters (NTs) is governed by both the frequency with which they engage the lipid bilayer (on‐rate) and the stability of these interactions once formed (off‐rate). As per previous analyses, to quantify these dynamics, we extracted association (on‐rate) and dissociation (off‐rate) constants from single‐molecule WGM spike signals, summarized in Figure S11 (Supporting Information). It can be seen that on‐rates vary across NTs, with dopamine and histamine exhibiting the lowest average binding frequencies (0.03 ± 0.003 s^−1^), while epinephrine shows the highest (0.070 ± 0.01 s^−1^). This trend suggests that amphiphilic NTs with greater hydrophobic character or more favorable membrane‐partitioning tendencies are more likely to encounter and engage with the bilayer surface. Conversely, the off‐rate variation highlights differences in interaction persistence: dopamine displays the lowest off‐rate (5.4 ± 0.59 s^−1^), indicating sustained membrane association, whereas L‐norepinephrine exhibits the highest (40.7 ± 2.66 s^−1^), consistent with more transient binding. These dissociation rates offer insights into molecular retention time within the membrane's optical near field, likely governed by molecular structure, charge distribution, and local lipid environment. Notably, while epinephrine exhibits both a high on‐rate and moderate off‐rate, suggesting frequent but relatively short‐lived interactions, dopamine's combination of moderate on‐rate and slow off‐rate indicates fewer but more stable binding events.

The step‐to‐spike ratio provides a complementary metric to kinetic analysis, capturing the likelihood that membrane‐binding events manifest as stable associations. While only loosely correlated with dissociation kinetics (off‐rates), the ratio aligns more closely with spike signal on‐rate behavior, reflecting the propensity of neurotransmitters to engage the membrane in favorable orientations that promote stable membrane partitioning. These findings suggest that the step‐to‐spike ratio can serve as a useful, qualitative readout of membrane‐binding efficiency, particularly when considered alongside kinetic parameters. These results highlight the complex and multi‐phase nature of aromatic NT interactions with lipid membranes and reinforce the critical role of membrane composition, NT structure, and molecular orientation in shaping synaptic signalling mechanisms.

Having established the kinetic signatures of NT interactions, we next examined how these rates vary with temperature to extract activation thermodynamic parameters using the Eyring equation:

(2) $$k=\frac{k_{B}T}{h}\exp\left(\frac{\Delta S^{‡}}{R}\right)\exp\left(-\frac{\Delta H^{‡}}{RT}\right)$$

Single‐molecule WGM sensing not only resolves individual binding and unbinding events of NTs at supported lipid bilayers but also enables extraction of kinetic and thermodynamic parameters when performed across a temperature series. By quantifying the temperature dependence of association and dissociation rates (only possible with spike‐like WGM signals, e.g., amino acid‐like NTs), *k*_*on*_ and *k*_*off*_, one can apply transition state theory via the Eyring equation to estimate activation enthalpies (Δ*H*‡), entropies (Δ*S*‡), and Gibbs free energies (Δ*G*‡) associated with membrane binding and desorption events. This approach combines single‐molecule kinetics with molecular energetics, providing insight into the strength and nature of NT–membrane interactions beyond equilibrium partitioning measurements such as ITC or SPR.

The Eyring analysis (Figure S13, Supporting Information) yields activation enthalpies for GABA dissociation from EggPC of ≈25–35 kJ mol^−1^ with small entropic terms (Δ*S*‡ ≈ −10 to +20 J mol^−1^ K^−1^), giving Δ*G*‡ ≈ 10–25 kJ mol^−1^ at 298 K. We estimated activation parameters from Eyring fits to the temperature dependence of *k*_off_, *k*_1on_, and *k*_2on_. Because the temperature span and the number of points are modest and the rate uncertainties are heterogeneous, the standard errors are large (Table S5, Supporting Information). We therefore interpret these thermodynamic parameters qualitatively, as they indicate weak, interfacial adsorption for hydrophilic NTs rather than precise quantitative values. Consistent with this, SPR studies on hydrophilic amino acid neurotransmitters report negligible binding energies on POPC membranes (Δ*G* ≈ 0 to −5, kJ mol^−1^; Δ*H* ≈ 0 kJ mol^−1^) and minimal partitioning.^[^ ^18^ ^]^ Taken together, the kinetic barriers and minimal equilibrium driving forces point to a model in which hydrophilic NTs interact only transiently and weakly with the bilayer interface.

## 3 Discussion

The results presented here represent, to the best of our knowledge, the first single‐molecule measurements of neurotransmitter (NT) interactions with supported lipid bilayers (SLBs), providing important new insights into the physicochemical basis of NT–membrane association. Our WGM platform demonstrated the resolution and sensitivity required to detect and differentiate transient versus stable NT–lipid interactions at nanomolar and attomolar scales. These capabilities allowed us not only to identify membrane‐active NTs, but to estimate their kinetic behaviors, interaction durations, and surface densities. Unlike ensemble techniques such as SPR, which often average out weak or transient binding events, our single‐molecule WGM platform captures individual NT–lipid interactions in real time. This sensitivity reveals both persistent and short‐lived association behaviors that would otherwise remain undetected, allowing for a more nuanced understanding of neurotransmitter–membrane dynamics. Our detection of binding at nM (and fM) concentrations, where prior ensemble methods report no signal, highlights the importance of single‐molecule approaches in studying membrane biophysics at physiologically relevant scales.

The distinction between spike‐like and step‐like resonance shifts enabled the functional classification of neurotransmitters into two broad groups: membrane‐active and membrane‐inert. NTs such as dopamine, histamine, L‐norepinephrine, and epinephrine produced frequent and stable step‐like events, indicating strong and sustained association with the lipid bilayer. In contrast, glycine, GABA, and glutamate produced predominantly spike‐like signals, reflecting short‐lived, low‐affinity interactions. The direct observation of stable membrane interactions for certain NTs supports the plausibility of a two‐phase targeting mechanism, in which some neurotransmitters first diffuse across the synaptic cleft (3D diffusion) and subsequently interact with membrane‐embedded receptors via lateral membrane diffusion. While our measurements do not capture lateral diffusion directly, the observed membrane residency times are consistent with a model in which the membrane serves as an intermediate or staging surface in neurotransmitter–receptor interactions. In contrast, membrane‐inert NTs likely access their receptors entirely through aqueous diffusion, targeting extracellular‐facing receptor domains.

This classification aligns closely with receptor biology. Membrane‐active NTs, such as histamine, dopamine, and serotonin, predominantly engage metabotropic receptors, including G‐protein coupled receptors (GPCRs), which are capable of binding ligands from within the lipid bilayer. Membrane‐inert NTs, such as glutamate and glycine, primarily act through ionotropic receptors that function via rapid extracellular signalling.^[^ ^2^ ^]^ This functional and physicochemical division may reflect evolutionary tuning of NT chemistry to optimize synaptic signalling pathways, balancing specificity, timing, and spatial control. It also suggests that membrane binding could play an underappreciated role in neurotransmitter localization, signalling kinetics, and receptor activation. Importantly, the measurements presented here were performed at nanomolar concentrations, significantly below those encountered in synaptic or intracellular environments.^[^ ^36^ ^]^ This implies that under physiological conditions, membrane accumulation of NTs may be significantly greater, potentially enabling the membrane to act as a localized reservoir that buffers, concentrates, or regulates NT availability near receptors. Our results therefore challenge traditional models of neurotransmission, which tend to focus solely on receptor‐bound signalling and neglect the role of membrane‐associated NTs. Quantitative analysis of WGM step‐like events provides surface density estimates of membrane‐associated NTs on the order of 10^12^ molecules cm^−2^ (Supporting Information). This range approaches prior SPR^[^ ^18^ ^]^ measurements on serotonin adsorption (4.4 × 10^13^ molecules cm^−2^), despite our experiments being conducted at concentrations six orders of magnitude lower. Previous studies using SPR reported no significant NT binding at concentrations below 10 millimolar, whereas our WGM platform clearly detected binding at 10 nanomolar. This difference underscores the advantage of single‐molecule techniques for detecting weak or transient interactions that are otherwise averaged out in ensemble methods.

To validate the WGM results, we performed constant‐area Langmuir trough experiments using DOPC monolayers. Histamine caused a significant and sustained increase in surface pressure (≈1.07 mN m^−1^), consistent with binding to the lipid interface, likely between the headgroup and acyl chain regions. Glycine, in contrast, produced only a modest shift (≈0.07 mN m^−1^), reinforcing the conclusion that its interactions are weak and non‐specific. Control injections of PBS produced a transient artifact that was accounted for in analysis. These ensemble‐level data strongly corroborate the single‐molecule findings, confirming that membrane association is NT‐specific and driven by defined molecular properties.

Further analysis revealed a consistent relationship between hydrophobicity, measured by LogP, and NT membrane affinity. While hydrophobicity, as reflected by LogP, offers a general indicator of membrane affinity, our findings highlight that other molecular features—such as charge distribution, size, and aromaticity—are equally critical in determining the strength and nature of NT–membrane interactions. Membrane‐active NTs such as dopamine (LogP = –0.98) and histamine (LogP = –0.70) exhibited longer dwell times and higher step‐to‐spike ratios, along with measurable pressure changes (histamine). More polar NTs, including GABA, glutamate, and glycine, produced low membrane association and minimal surface pressure effects (glycine). Notably, glycine, the most hydrophilic NT tested, showed a slightly longer dwell time than GABA and glutamate, suggesting that size, conformational flexibility, or charge distribution may also modulate transient interactions. Taken together, these results identify aromaticity, amphiphilicity, and charge neutrality as key molecular features promoting membrane association, and support the hypothesis that membrane engagement is a selective and structured process.

By combining high‐resolution WGM sensing with Langmuir trough measurements, we demonstrate a coherent approach for understanding NT–membrane interactions. This study shows that single‐molecule optical sensing can reveal transient and heterogeneous membrane interactions that are invisible to ensemble methods. By resolving individual neurotransmitter–lipid binding events with nanomolar sensitivity, our platform provides a new window into the biophysical underpinnings of synaptic transmission. These findings suggest that lipid membranes are not simply passive barriers, but active modulators of synaptic signalling, capable of concentrating or excluding neurotransmitters in ways that directly affect receptor access and activation. More broadly, this highlights the potential of single‐molecule techniques to uncover non‐canonical modes of neurotransmitter action that are modulated at the membrane level.

This study provides compelling evidence that neurotransmitters interact with lipid membranes in chemically selective and biologically relevant ways. Membrane‐active NTs such as histamine, epinephrine, and dopamine partition into lipid bilayers, likely contributing to spatially regulated receptor targeting, particularly for GPCRs embedded within the membrane. Membrane‐inert NTs such as glycine, GABA, and glutamate show minimal interaction with the bilayer, consistent with their rapid, aqueous‐phase access to ionotropic receptors. The WGM platform's ability to detect membrane association at nanomolar concentrations offers new insight into NT behavior at physiologically relevant levels, revealing interaction modes that conventional techniques overlook. These findings support a revised model of neurotransmission in which membrane engagement is an integral aspect of NT localization, receptor access, and signal modulation.

Moving forward, studies investigating lipid composition, membrane charge, and the presence of membrane‐embedded NT receptors will be essential to further unravel the mechanistic basis of NT–lipid association. In particular, cholesterol is expected to significantly modulate NT–membrane kinetics by condensing the PC matrix (reducing area per lipid, increasing bilayer order), thickening the membrane, and raising the interfacial dipole potential. For hydrophilic amino‐acid NTs, these changes would likely suppress adsorption by reducing interfacial water penetration and packing defects, leading to fewer spike events (lower *k*_on_) and slightly faster disengagement (higher *k*_off_).

For aromatic NTs such as dopamine, which both literature and our data place at the headgroup–water interface, cholesterol is expected to reduce overall partitioning by decreasing free area and headgroup fluctuations. At the same time, any cholesterol‐induced increase in dipole potential could weakly stabilize cationic headgroups near the surface, modestly prolonging residence times for certain binding geometries. In phase‐separating mixtures (PC/SM/Chol), we would therefore anticipate preferential partitioning of aromatic NTs into disordered *L*_*d*_ domains and a sharper selectivity between hydrophilic and aromatic species at domain boundaries: fewer capture events in *L*_*o*_ (cholesterol‐rich) regions, more in *L*_*d*_, and possibly a shift in TE/TM anisotropy toward interfacial rather than deeply inserted orientations. Overall, increasing cholesterol would be expected to decrease step‐event frequency for aromatic NTs while further suppressing spikes from hydrophilic NTs, thereby preserving, or perhaps enhancing, the relative selectivity for aromatic NTs.

Future investigations incorporating membrane‐embedded receptors could enable direct observation of receptor‐mediated interactions, providing a deeper understanding of the molecular mechanisms underlying synaptic transmission. With continued technical improvements such as increased sensitivity and higher temporal resolution, which remain attainable given that current measurements are not yet limited by fundamental device constraints, this approach may ultimately resolve subtle signals associated with conformational changes in membrane‐embedded receptors. This would enable the investigation of receptor‐mediated neurotransmission at the single‐molecule level, including systems involving serotonin and its interaction with receptors such as 5‐HT2A.^[^ ^37^ ^]^ Such studies could help clarify the role of the membrane in modulating receptor function and advance our understanding of how psychoactive compounds and therapeutics influence neural signalling, with broad relevance for mental health research. Incorporating membrane models with defined domain structures or receptor‐functionalized interfaces could clarify the structural requirements for NT intercalation and its physiological relevance. These findings also support the view that lipid membranes may act as dynamic reservoirs for neurotransmitters, transiently retaining or concentrating them at the membrane interface. This sequestration could influence neurotransmitter availability, synaptic dwell time, and the spatial fidelity of receptor activation, effectively shaping the background level of chemical signalling.

To provide a direct experimental link between partitioning dynamics and receptor activation, we propose that single‐molecule WGM experiments on membranes preloaded with neurotransmitters and functionalized with receptors could simultaneously track interfacial binding events and receptor activation kinetics. Such measurements would test whether membrane partitioning acts to pre‐concentrate ligands near receptors, thereby modulating local neurotransmitter availability and synaptic signalling efficiency. Intriguingly, this model also raises the possibility that anesthetics might operate, at least in part, by displacing endogenous neurotransmitters from membrane association sites, altering local NT concentrations and thus modulating neuronal responsiveness. Altogether, this work strengthens the hypothesis that neurotransmitters interact with the membrane in selective and dynamic ways, suggesting that the lipid environment is not just a backdrop but a functionally active component in synaptic communication.

## 4 Experimental Section

WGM resonators were fabricated by melting a single‐mode optical fibre (SMF 2e8, Corning GmbH, Germany) with a 30 W CO_2_ laser (Synrad 482, Novanta Inc., USA) using a home‐built set‐up, to make silica microspheres of ≈80‐90 µm in diameter. WGMs were excited using an external cavity diode laser (λ = 780 nm, Toptica, Germany) focused onto a prism coupler (N‐SF11), with a V‐shaped polydimethylsiloxane (PDMS) chamber connected to a glass coverslip serving as a loading chamber. The raw WGM spectra were obtained every 20 ms using swept‐laser scanning and recorded by a custom LabVIEW programme, tracking the resonance peak position (Δλ) using a centroid fitting algorithm23. Full‐width‐at‐half‐maximum (FWHM), referring to the linewidth (κ), was determined directly from the spectrum by fitting the resonant mode with a Lorentzian.

Liposomes were fabricated using the thin film hydration method.^[^ ^38^ ^]^ EggPC (840051C‐1G, Avanti Polar Lipids, Alabaster, AL, USA) and cholesterol (C8667‐1G, Sigma‐Aldrich, St. Louis, MO, USA) were combined to make an ≈10% cholesterol solution. The solution was dried with nitrogen gas, and subsequently desiccated in a vacuum desiccator. The lipid mixture was rehydrated with 8 mL of filtered PBS and vortexed for 1 min, ensuring sufficient mixing. One milliliter of the sample was extruded through a 100 nm filter (Avanti Polar Lipids, Alabaster, AL, USA) 11 times to produce unilamellar liposomes of an appropriate size. Fifty microliters of the extruded liposome solution was diluted and quality checked using a Zetasizer (Malvern, UK), ensuring uniform liposome size across all experiments (Figure S1, Supporting Information). The liposomes were stored at 4°C.

The glass microspheres were functionalized with Cetyl‐triammonium bromide (CTAB) gold nanorods (10 × 35 nm, A1210‐780‐CTAB, Nanopartz Inc., USA in pH 1.6 HCl (300 µL, 24 mM). Each binding event was recognized as an observable step in the Δλ and FWHM traces. After observation of ≈200 fm shift in the FWHM trace (corresponding to the addition of ≈4 NRs), the solution was removed and the chamber was rinsed with Milli‐Q water, prior to the addition of PBS (300 µL) and the liposomes (60 µL). This chamber solution was left to incubate for 1 hour minimum, to assure full bilayer coverage (See Supporting Information). The glycine (MW = 75.07 gmol^−1^, G7126‐100G, Sigma–Aldrich) and epinephrine hydrochloride (MW = 129.67 gmol^−1^, E4642‐25G, Sigma–Aldrich) experiments were performed separately, following the same preparation procedure. After stock solutions (1 mM) were made for each NT with PBS, serial dilutions were performed to achieve the initial NT concentrations: 10 pM, 10 nM, and 1 mM (epinephrine), and 1 mM, 10 mM, and 100 mM (glycine). After liposome incubation, the solution was removed and the chamber was rinsed three times with both Milli‐Q water and PBS, before adding 300 µL of PBS. The pH for the aromatic NTs was kept at 6.5 as higher pH resulted in NT polymerization, ultimately contaminating the chamber and rendering measurements impossible. For the amino‐acid types NTs the pH was 7.4. Once the background was recorded for 5 min, 3 µL of the chamber solution was replaced with the lowest NT concentration (i.e. 10 pM or 1 mM) and the spectra were recorded for 15 min. This was repeated, following increasing concentration, for the subsequent two solutions, resulting in final chamber concentrations of 100 fM, 100 pM, and 10 nM (aromatic NTs) and 10 nM, 1 mM, and 100 mM (amino acid NTs). A pipette was used to inject and mix chamber solutions each time.

Monolayers of 1,2‐dioleoyl‐sn‐glycero‐3‐phosphocholine (DOPC) were prepared on a Langmuir trough (Kibron MicroTrough S) filled with 60 mL of phosphate‐buffered saline (PBS). A 10 µL aliquot of DOPC dissolved in chloroform (10 mg mL^−1^) was carefully spread onto the subphase. DOPC was selected for Langmuir monolayer experiments because it is a single, well‐defined lipid species, avoiding the natural heterogeneity of EggPC and ensuring superior trough reproducibility. Its choline–phosphate headgroup chemistry and mixed acyl chain character yield interfacial properties comparable to EggPC, allowing meaningful comparison of headgroup interactions across techniques. Cholesterol was deliberately omitted in monolayers to isolate lipid–NT interactions. After spreading, the barrier separation was adjusted to achieve a surface pressure of 30 mN m^−1^, and the monolayer was left for one hour to equilibrate. Once stable, neurotransmitters were injected directly into the subphase with minimal disturbance to the interface. Surface pressure was continuously recorded following injection to monitor changes in membrane packing and interactions over time.

## Conflict of Interest

The authors declare no conflict of interest.

## Author Contributions

T.L.D. and A.K.A. contributed equally to this work. T.L.D. and A.K.A. designed and performed the experiments and analyzed the data. R.C. and E.S. conducted WGM measurements. All authors contributed to writing and editing the manuscript.

## Supporting information

Supporting Information

## Data Availability

The data that support the findings of this study are available on request from the corresponding author. The data are not publicly available due to privacy or ethical restrictions.

## References

[1] T. M. Jessell , S. A. Siegelbaum , A. J. Hudspeth , E. R. Kandel , J. H. Schwartz , Principles of Neural Science, Fifth Edition, McGraw‐Hill Education, New York, NY, 2014.

[2] P. A. Postila , I. Vattulainen , T. Róg , Sci. Rep. 2016, 6, 19345. 26782980 10.1038/srep19345 PMC4725992

[3] F. Lolicato , H. Juhola , A. Zak , P. A. Postila , A. Saukko , S. Rissanen , G. Enkavi , I. Vattulainen , M. Kepczynski , T. Róg , ACS Chem. Neurosci. 2020, 11, 1914. 32538079 10.1021/acschemneuro.9b00656 PMC7735663

[4] C. Wang , F. Ye , G. F. Velardez , G. H. Peters , P. Westh , J. Phys. Chem. B 2011, 115, 196. 21158460 10.1021/jp108368w

[5] P. Parkkila , T. Viitala , ACS Chem. Neurosci. 2020, 11, 969. 32101397 10.1021/acschemneuro.0c00049 PMC7145343

[6] S. Das , P. Purkayastha , Langmuir 2017, 33, 7281. 28661681 10.1021/acs.langmuir.7b01173

[7] A. K. Sahu , A. K. Mishra , Langmuir 2021, 37, 13430. 34732050 10.1021/acs.langmuir.1c02184

[8] G. H. Peters , M. Werge , M. N. Elf‐Lind , J. J. Madsen , G. F. Velardez , P. Westh , Chem. Phys. Lipids 2014, 184, 7. 25159594 10.1016/j.chemphyslip.2014.08.003

[9] A. Shafieenezhad , S. Mitra , S. R. Wassall , S. Tristram‐Nagle , J. F. Nagle , H. I. Petrache , Biophys. J. 2023, 122, 1118. 36804668 10.1016/j.bpj.2023.02.016 PMC10111280

[10] Y. Matam , B. D. Ray , H. I. Petrache , Neurosci. Lett. 2016, 618, 104. 26960008 10.1016/j.neulet.2016.02.052

[11] K. Jodko‐Piorecka , G. Litwinienko , ACS Chem. Neurosci. 2013, 4, 1114. 23662798 10.1021/cn4000633 PMC3715842

[12] B. Biswas , P. C. Singh , J. Phys. Chem. Lett. 2021, 12, 2871. 33720729 10.1021/acs.jpclett.1c00173

[13] A. Mugler , F. Tostevin , P. R. ten Wolde , Proc. Natl. Acad. Sci. USA 2013, 110, 5927. 23530194 10.1073/pnas.1218301110 PMC3625283

[14] R. S. Cantor , Biochemistry 2003, 42, 11891. 14556619 10.1021/bi034534z

[15] I. Alves , G. Staneva , C. Tessier , G. F. Salgado , P. Nuss , Biochim. Biophys. Acta, Biomembr. 2011, 1808, 2009. 10.1016/j.bbamem.2011.02.021 21377444

[16] A. H. Hansen , K. T. Sørensen , R. Mathieu , A. Serer , L. Duelund , H. Khandelia , P. L. Hansen , A. C. Simonsen , Chem. Phys. Lipids 2013, 175‐176, 84. 10.1016/j.chemphyslip.2013.08.002 23994552

[17] H. Jerabek , G. Pabst , M. Rappolt , T. Stockner , J. Am. Chem. Soc. 2010, 132, 7990. 20527936 10.1021/ja910843d

[18] B. P. Josey , F. Heinrich , V. Silin , M. Lösche , Biophys. J. 2020, 118, 1044. 32032504 10.1016/j.bpj.2020.01.016 PMC7063487

[19] M. Robinson , S. Turnbull , B. Y. Lee , Z. Leonenko , Biochim. Biophys. Acta, Biomembr. 2020, 1862, 183363. 32450141 10.1016/j.bbamem.2020.183363

[20] Y. Huang , P. Chen , L. Zhou , J. Zheng , H. Wu , J. Liang , A. Xiao , J. Li , B.‐O. Guan , Adv. Mater. 2023, 35, 2304116. 10.1002/adma.202304116 37342974

[21] S. Frustaci , F. Vollmer , Curr. Opin. Chem. Biol. 2019, 51, 66. 31202140 10.1016/j.cbpa.2019.05.003

[22] D. Yu , M. Humar , K. Meserve , R. C. Bailey , S. N. Chormaic , F. Vollmer , Nat. Rev. Methods Primers 2021, 1, 83.

[23] M. Loyez , M. Adolphson , J. Liao , L. Yang , ACS Sens. 2023, 8, 2440. 37390481 10.1021/acssensors.2c02876

[24] M. C. Houghton , N. A. Toropov , D. Yu , S. Bagby , F. Vollmer , Adv. Sci. 2024, 11, 2403195. 10.1002/advs.202403195 PMC11425209 38995192

[25] E. Kim , M. D. Baaske , I. Schuldes , P. S. Wilsch , F. Vollmer , Sci. Adv. 2017, 3, e1603044. 28435868 10.1126/sciadv.1603044 PMC5371424

[26] S. Subramanian , H. B. Jones , S. Frustaci , S. Winter , M. W. van der Kamp , V. L. Arcus , C. R. Pudney , F. Vollmer , ACS Appl. Nano Mater. 2021, 4, 4576. 34085031 10.1021/acsanm.1c00176 PMC8165693

[27] S. Vincent , S. Subramanian , F. Vollmer , Nat. Commun. 2020, 11, 2043. 32341342 10.1038/s41467-020-15822-8 PMC7184569

[28] J. Topolancik , F. Vollmer , Appl. Phys. Lett. 2006, 89, 184103.

[29] A. K. Arunkumar , J. Xavier , F. Vollmer , in Frontiers in Optics + Laser Science 2023 (FiO, LS) . Optica Publishing Group, Washington, DC 2023 JTu5A.1, https://opg.optica.org/abstract.cfm?URI=FiO‐2023‐JTu5A.1.

[30] A. K. Arunkumar , E. Zossimova , M. Walter , S. Pedireddy , J. Xavier , F. Vollmer , arXiv 2025, https://arxiv.org/abs/2507.10146.

[31] M. A. McCloskey , M. Poo , J. Cell Biol. 1986, 102, 88. 3001105 10.1083/jcb.102.1.88 PMC2114064

[32] G. Vauquelin , A. Packeu , Mol. Cell. Endocrinol. 2009, 311, 1. 19647036 10.1016/j.mce.2009.07.022 PMC7116919

[33] T.‐H. Lee , K. N. Hall , M. J. Swann , J. F. Popplewell , S. Unabia , Y. Park , K.‐S. Hahm , M.‐I. Aguilar , Biochim. Biophys. Acta, Biomembr. 2010, 1798, 544. 10.1016/j.bbamem.2010.01.014 20100457

[34] A. Bianchetti , A. Federico , S. Vincent , S. Subramanian , F. Vollmer , Opt. Commun. 2017, 394, 152.

[35] P. A. Postila , T. Róg , Mol. Neurobiol. 2020, 57, 910. 31595461 10.1007/s12035-019-01775-7 PMC7031182

[36] A. Scimemi , M. Beato , Mol. Neurobiol. 2009, 40, 289. 19844813 10.1007/s12035-009-8087-7 PMC2777263

[37] I. Raote , A. Bhattacharya , M. Panicker , in Serotonin Receptors in Neurobiology , (Ed.: A. Chattopadhyay ) chapter 6. CRC Press/Taylor & Francis, Boca Raton, FL 2007, https://www.ncbi.nlm.nih.gov/books/NBK1853/.

[38] M. J. Hope , M. B. Bally , G. Webb , P. R. Cullis , Biochim. Biophys. Acta, Biomembr. 1985, 812, 55. 10.1016/0005-2736(85)90521-8 23008845

