# Supplementary material for: Uncovering the Lipid Interface in Neurotransmission: Single Molecule Measurements of Neurotransmitters Interacting with Membranes Reveal Species Dependent Membrane Binding
Source: Adv Sci (Weinh). 2025 Nov 12;13(11):e15727. doi: 10.1002/advs.202515727 (PMC12931208; doi:10.1002/advs.202515727)
Supplement: Supplementary file 1 — Supporting Information [file ADVS-13-e15727-s001.pdf]

Supplementary Information: Uncovering the Lipid Interface in Neurotransmission: Single molecule measurements of neurotransmitters interacting with membranes reveal species dependent membrane binding

Thomas L. Derrien[1,*,+], Aneeth Kakkanattu Arunkumar[1,*,+], Rosalind Cross[1], Emilie Sunnucks[1], and Frank Vollmer[1]

[1]Department of Physics and Astronomy, Living Systems Institute, University of Exeter, Exeter, UK

[*]corresponding.author@email.example

[+]these authors contributed equally to this work

October 1, 2025

Supplementary information

Estimation of the size of unilamellar vesicles

The size of the unilamellar vesicles was determined by dynamic light scattering (DLS) on a Zetasizer (Malvern Instruments, UK). A sample consisting of 50 μl of the extruded liposome suspension was diluted in 200 μl of phosphate-buffered saline (PBS) prior to analysis. This assessment was performed to confirm appropriate bilayer thickness and uniformity. The resulting size distribution profile of the liposomes is presented in Figure 1. The extruded liposomes, passed through a 100 nm polycarbonate filter, exhibited an average diameter of 125.6 nm, falling within the expected distribution range. These results indicate the successful formation of unilamellar liposomes.

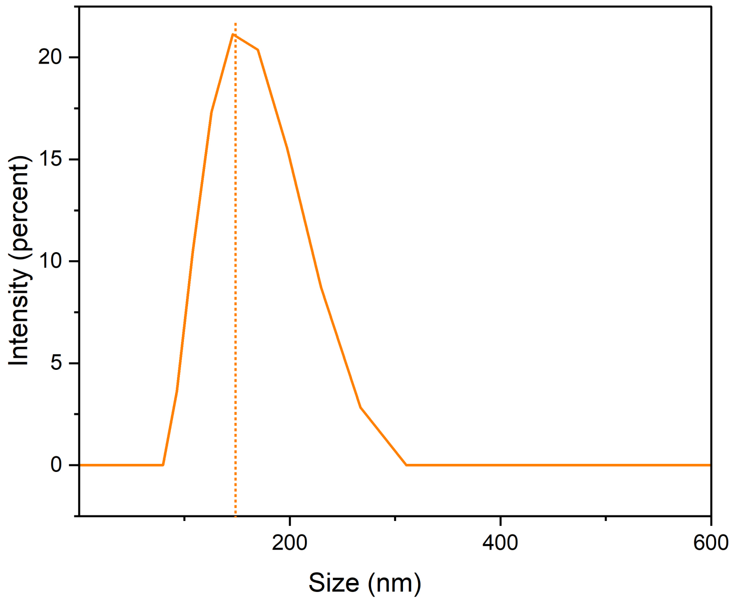

Figure 1: **Intensity distribution of liposome from zeta sizer**. The liposomes are approximately 125.6 nm in diameter

Estimation of lipid coverage

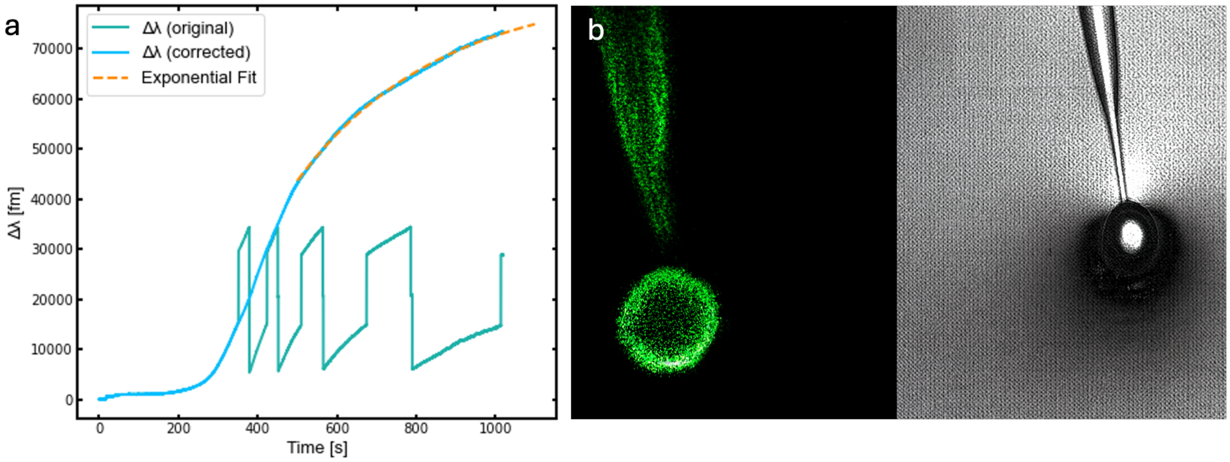

Figure 2: a) WGM shift upon addition of liposomes. The plot shows the original data (green) whose trace shows sudden shifts when a mode moves out of the scan range, and the tracking software moves to another mode. These jumps are removed in the corrected data (blue) to enable simple estimation of the saturation shift from an exponential fit (orange). b) Confocal fluorescence microscopy images demonstrating bilayer coverage on the microsphere surface. The lipid bilayer was labelled with 0.5% fluorescent lipid probe (18:1 NBD-PE, Avanti Polar Lipid, USA), and the resulting images show continuous fluorescence around the equatorial region of the microsphere, indicating uniform membrane coverage.

To estimate the extent of lipid adsorption on the microsphere surface, we monitored the WGM shift upon incubation of liposomes. The lipid coverage can be estimated using the resonance condition for whispering gallery modes (WGMs), which relates the resonance wavelength λ to the refractive index n and radius R of the sphere:

$$m\lambda = 2\pi n R \tag{1}$$

Assuming constant refractive index and mode number, a shift in resonance wavelength can be directly related to a change in the sphere's radius:

$$\frac{d\lambda}{\lambda} = \frac{dR}{R} \tag{2}$$

$$\frac{\Delta\lambda}{\lambda} \approx \frac{\Delta R}{R} \quad \Rightarrow \quad \Delta R \approx R \cdot \frac{\Delta\lambda}{\lambda} \tag{3}$$

By applying this relation, and using the asymptotic value obtained from fitting an exponential model to the corrected resonance shift data (Figure 2), we estimated the effective change in radius due to lipid adsorption.

The microsphere had a diameter of 80 μm and the WGM was excited with a 785 nm laser. The exponential fit yielded a total shift of $\Delta\lambda$ = 82,199 fm. This corresponds to an estimated increase in radius of approximately **4.1 nm**, which likely represents the saturation coverage of lipids on the glass surface. This value is approximately the thickness of a lipid bilayer, confirming the lipid bilayer assembly onto the WGM sensor.

Event Detection and Classification

Time-resolved WGM traces were processed using a custom MATLAB (MathWorks, USA) graphical user interface designed for automated event detection and classification (https://github.com/ssubr[illegible] were first corrected for slow thermal or mechanical drift using a first-order Savitzky–Golay filter (window length set according to the sampling rate) to preserve millisecond-scale kinetics while removing baseline fluctuations.

Following drift correction, single-molecule events were identified using a two-stage procedure:

1. **Event detection:** Individual events were detected using the MATLAB `findpeaks` function with an adaptive threshold set at 3–5σ, where σ is the standard deviation of the baseline noise. The noise level was estimated by dividing the trace into non-overlapping windows (N = 500–1000 points) and calculating the minimum variance across all windows to avoid contamination by real events. Thresholds above 5σ did not alter event counts, confirming low false-positive rates.

2. **Event classification:** Detected events were classified as *spikes* or *steps* based on dwell-time analysis. Transient events returning to baseline within the acquisition window were labeled as spikes, while events persisting for the remainder of the trace were labeled as steps.

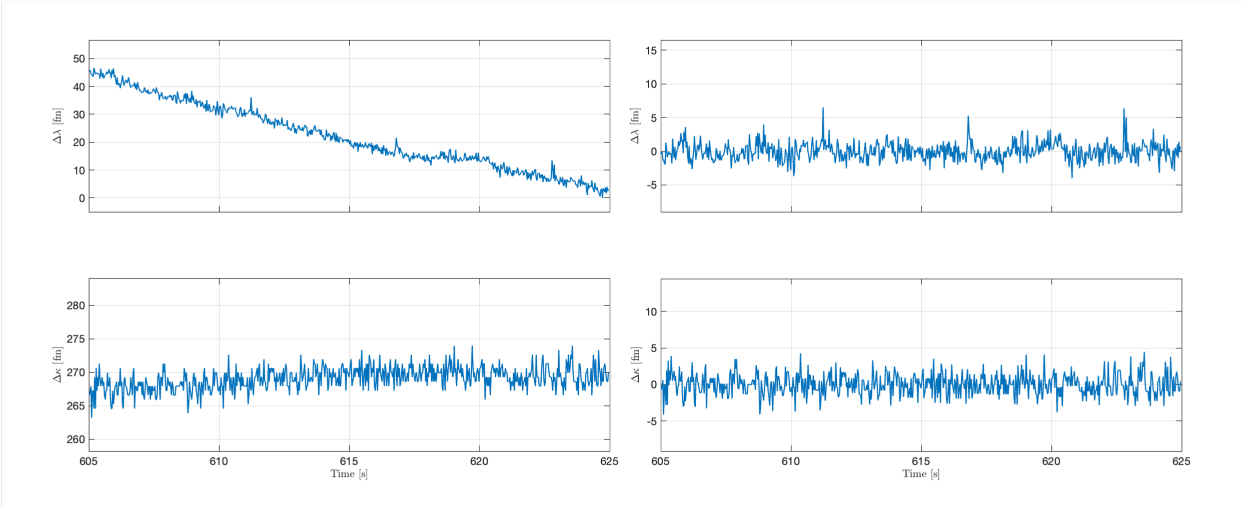

Figure 3: Representative traces of spike-like signals originating from 1 μM GABA before (left) and after (right) drift correction.

Survival analysis

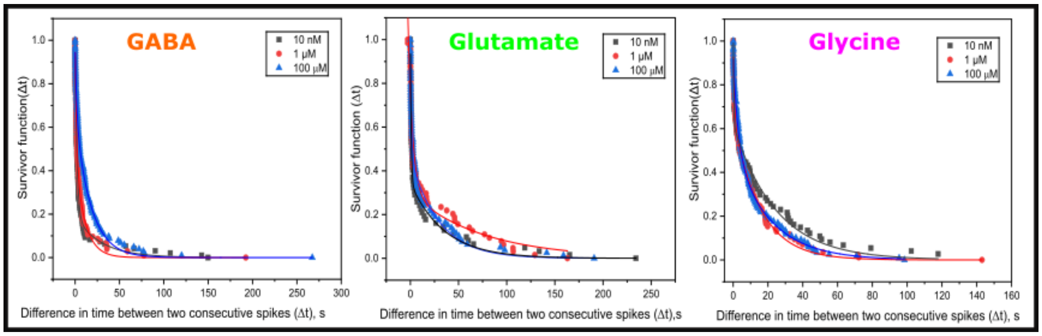

Figure 4: **Survival plot of time interval between spike events for GABA, glutamate, and glycine.**

Interactions with membrane non-binding neurotransmitters-with and without membrane

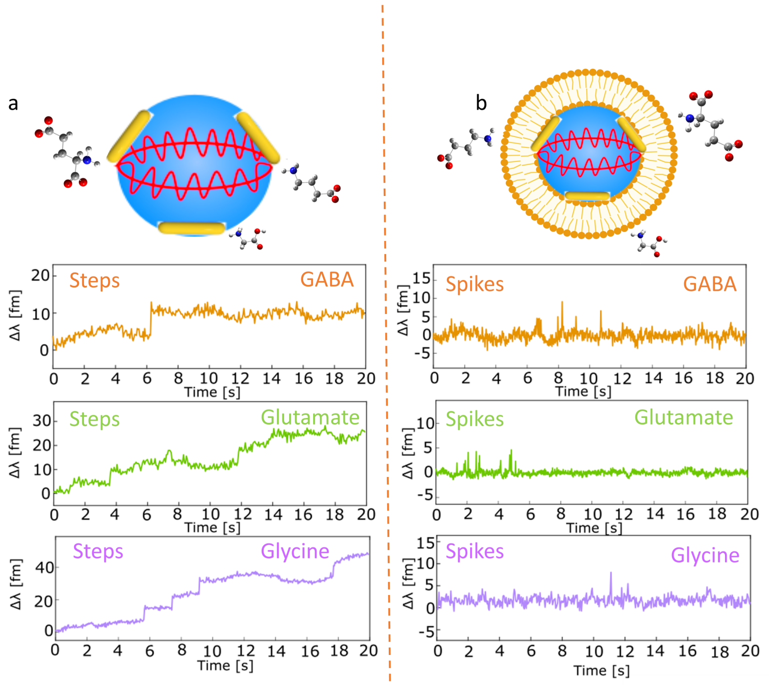

Figure 5: **Interaction of GABA, glutamate and glycine with WGM sensors with and without membrane.** **a**, Interaction of GABA, glutamate and glycine with optoplasmonic WGM sensor forming step signals without membrane**b**, Interaction of GABA, Glutamate and Glycine with optoplasmonic WGM sensor with membrane, obtaining spike signals.

To verify that the observed optoplasmonic whispering-gallery mode (WGM) signals originated specifically from neurotransmitter–membrane interactions, control experiments were

conducted under identical conditions, with and without the presence of a lipid bilayer. Experimental parameters included the use of microspheres with a diameter of 89 μm, each functionalized with 3–4 gold nanorods (AuNRs), and exposure to 10 nM concentrations of neurotransmitters in 40 mM phosphate-buffered saline (PBS), as was previously done in phosphate buffer [1,2]. As shown in Figure 3, in the absence of the lipid membrane, neurotransmitters such as γ-aminobutyric acid (GABA), glutamate, and glycine elicited step-like WGM signals upon interaction with the AuNRs. These step responses are attributed to the formation of hydrogen bonds between the neurotransmitters (due to their protonated amines in the structure) and the chemisorbed phosphate anions on the AuNR surfaces.

In contrast, when the lipid bilayer was present, the same neurotransmitters generated distinct spike-like signals under otherwise identical conditions. These transient spikes are indicative of repulsive interactions, likely arising from lipophobicity of the neurotransmitters with respect to the lipid membrane. The spike-like WGM sensograms are characteristic of membrane non-binding neurotransmitter interactions with the lipid bilayer, thereby confirming the differential sensitivity of the optoplasmonic sensor to membrane-associated events compared to the step events in the absence of membrane.

Histogram distributions of membrane binding NTs at various concentrations

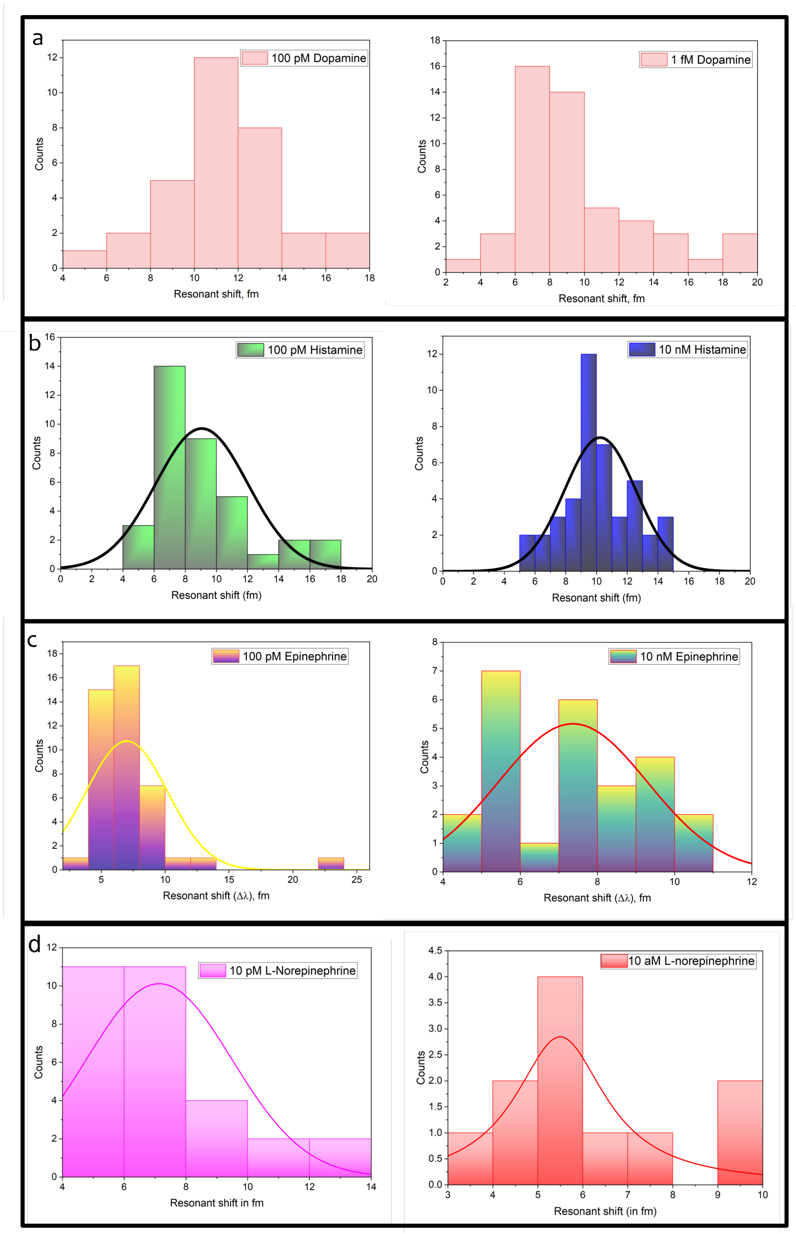

Figure 6: [(A) Dopamine at 100 pM (left) and 1 fM (right); (B) Histamine at 100 pM (left) and 10 nM (right); (C) Epinephrine at 100 pM (left) and 10 nM (right); (D) L-Norepinephrine at 10 pM (left) and 10 aM (right). Histograms show the distribution of WGM resonance wavelength shifts (in femtometers) induced by single-molecule binding events at the microsphere surface.

Control experiments showing the origin of step signals from the membrane binding neurotransmitters

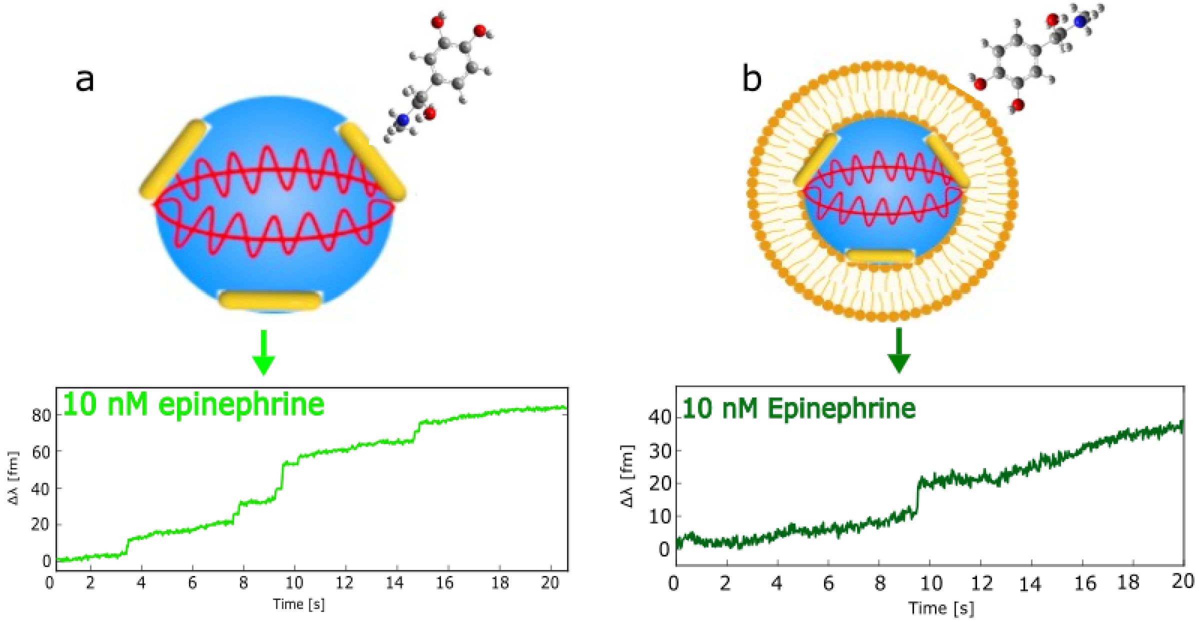

Figure 7: Signals obtained from the epinephrine **a**, without membrane (light green) and **b**, with membrane (dark green). Step signals are observed from epinephrine binding with AuNRs and epinephrine binding with the lipid bilayers coated on a nanorod-modified microsphere. The experiments were carried out at pH 6.5 in PBS at 10 nM concentration of epinephrine.

Membrane-binding neurotransmitters predominantly recorded step signals from the membrane, as we previously showed for dopamine, histamine and L-norepinephrine [1,2]. However, it was also observed previously that these neurotransmitters could directly bind to the nanorods, forming step signals (without the membrane) as shown in Figure 7a. Therefore, if the lipid bilayers do not have complete coating coverage on the nanorod-modified microsphere, the neurotransmitters could bind to the exposed gold nanorod and give step signals. Hence, confirmation is necessary that the step signals observed from membrane-binding neurotransmitters are due to interactions with the lipid bilayers.

Notably, in addition to step-like signals, spike-like transients are also observed upon interaction of membrane-binding neurotransmitters with lipid bilayers. However, these neurotransmitters are unable to generate spike signals directly upon contact with gold nanorods, as the pKa of their amine groups exceeds the experimental pH of 6.5, preventing effective binding. As a result, spike signals are uniquely attributable to membrane interactions. Therefore, this control experiment primarily focuses on evaluating the legitimacy of the step-like signals arising from membrane-binding neurotransmitters.

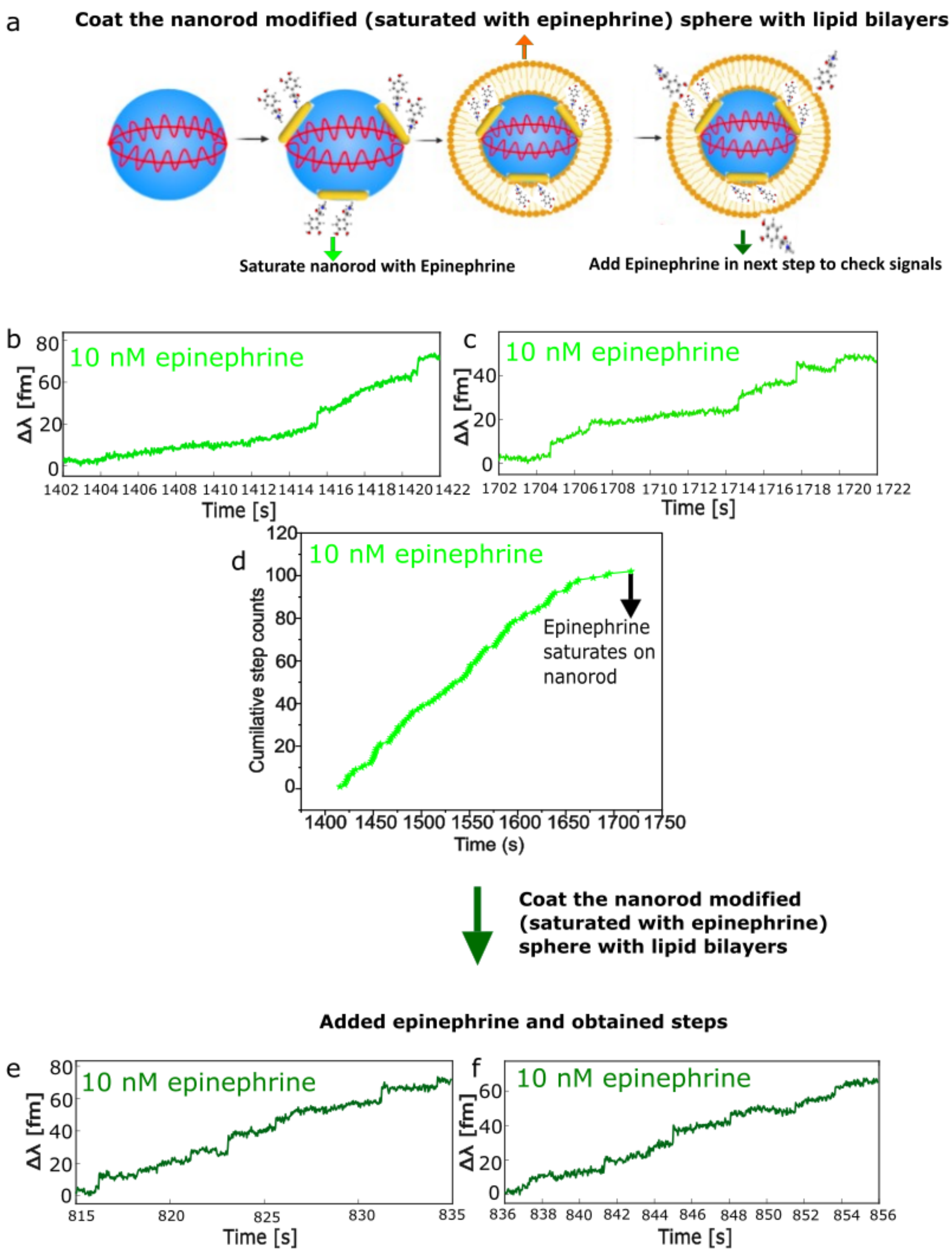

Figure 8: **Control experiment showing the origin of step signals by membrane binding neurotransmitters.** **a**, Schematic of the control experiment. **b-c**, Step signals at different time intervals (light green) when epinephrine binds with nanorods at different time traces and saturates the membrane.**d**, saturation curve from the binding step events of 10 nM epinephrine saturating the nanorods. **e-f**, Step events (dark green) at different time traces when epinephrine interacts with lipid bilayer coated sensor assembly. The times shown are relative to the start of the experiment.

To validate the origin of the step-like signals attributed to membrane-binding neurotransmitters, a control experiment was conducted in which the gold nanorods were saturated with neurotransmitters. Epinephrine, representative of the membrane-binding class, was used for this experiment under the assumption that similar behaviour would be exhibited by the other neurotransmitters in this group.

The schematic of the experiment is illustrated in Figure 8a. Following sensor assembly, the gold nanorods were saturated with 10 nM epinephrine, effectively occupying all available sensing sites and thereby preventing further step-like events at pH 6.5. A concentration of 10 nM was deliberately selected to ensure sufficient surface coverage for saturation. Notably, higher concentrations of epinephrine are avoided due to their propensity for polymerization. Hence, an optimum concentration level of 10 nM is chosen to obtain sufficient concentration to saturate the nanorods and avoid the polymerization of the molecules simultaneously. Step-like signals recorded at different time intervals upon direct binding of epinephrine to gold nanorods in the absence of lipid bilayers are presented in Figure 8b,c. The experiment was carried out over a 30-minute duration to ensure complete saturation of the nanorod surface, beyond which no further step events were observed. Saturation is further confirmed by the cumulative event count plotted over time in Figure 8d, where the curve exhibits a plateau, indicating the cessation of binding events. All data corresponding to epinephrine–nanorod interactions are shown in light green for clarity.

Following saturation of the nanorods with epinephrine, the sensor assembly was incubated with liposomes at pH 7.3 to form lipid bilayers around the nanorods. The chamber was subsequently washed and refilled with phosphate-buffered saline (PBS) at pH 6.5, after which 10 nM epinephrine was reintroduced. The reappearance of step-like signals under these conditions confirms clearly shows that the observed events originate from the interaction of epinephrine with the lipid membrane rather than with the nanorods, which were already saturated. Representative traces of these membrane-originated step signals are shown in Figure 8e,f, highlighted in dark green.

These results underscore that the step-like signals obtained from the membrane-binding neurotransmitters are due to their interaction with the membrane, rather than with the gold nanorods. This establishes the capability of optoplasmonic WGM sensors as powerful platforms for probing the dynamic interplay between neurotransmitters and membranes. To the best of our knowledge, this represents the first quantitative interpretation of such complex interactions—processes that are fundamental to neuronal function and synaptic transmission.

Step Interval Suvivor Functions

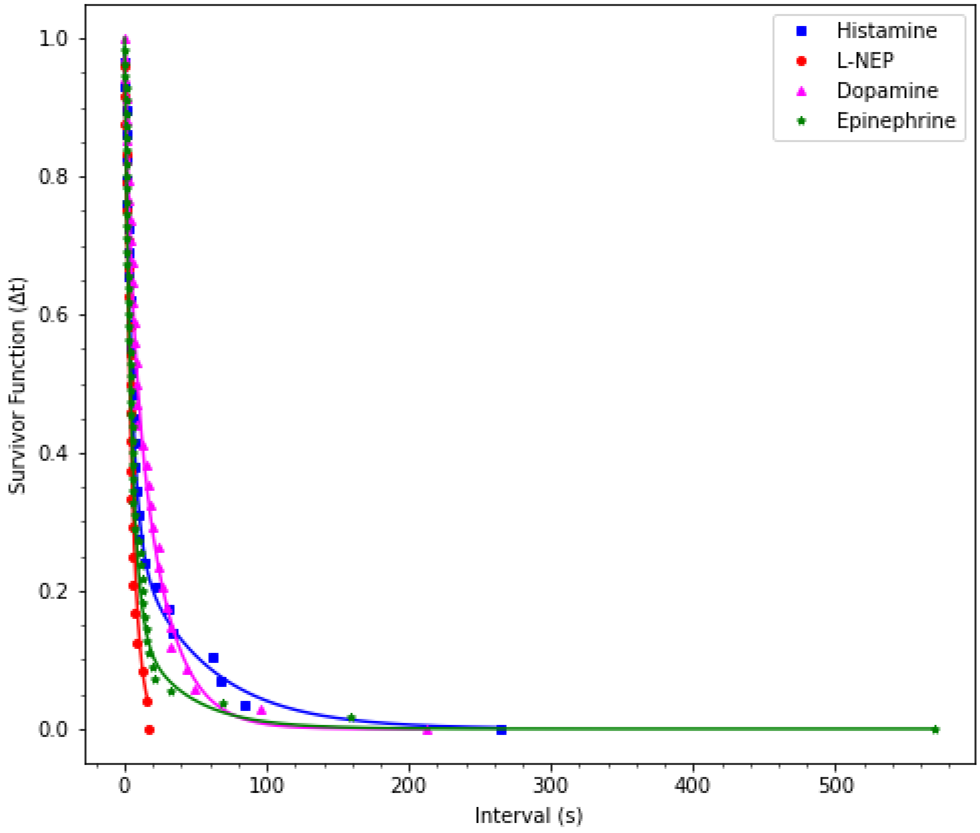

Figure 9: Step interval survivor functions for membrane-binding neurotransmitters at 100 fM. Step-to-step time intervals (Δt) were analyzed using survival analysis for Histamine, L-norepinephrine (L-NEP), Dopamine, and Epinephrine. The empirical survivor data (symbols) were fit using double-exponential models (solid lines), revealing bi-exponential kinetics for all compounds except L-NEP, which was best described by a single-exponential fit. These results support the presence of a dimensionality shift in membrane association dynamics for most neurotransmitters, consistent with a two-phase binding process involving both 3D diffusion from solution and 2D membrane surface diffusion.

Polarisation Anisotropy

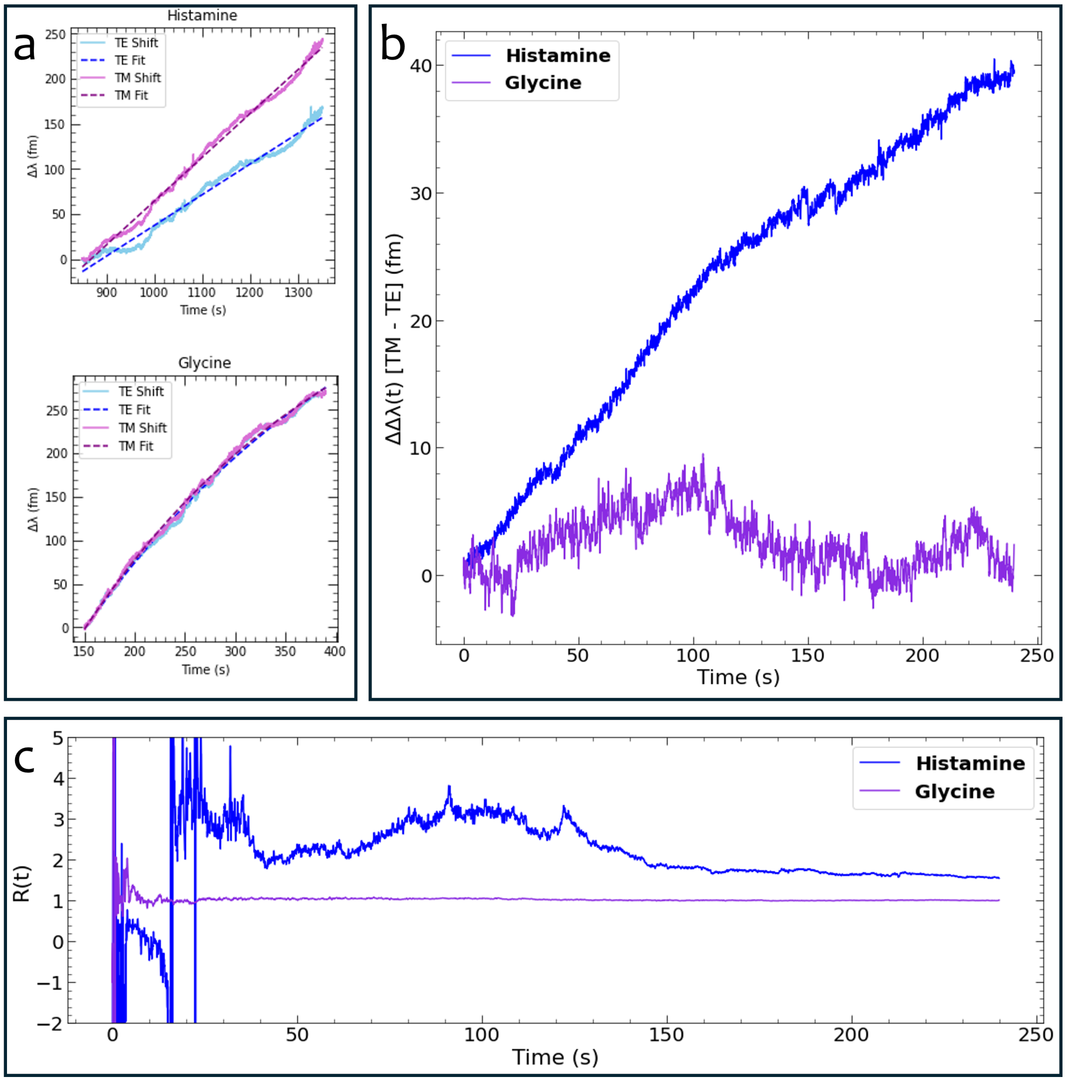

Figure 10: Resonance shifts measured in TE and TM modes indicate differences in the axial vs. radial electric field response. TE and TM mode shift time traces (A). Differince in mode shifts ($\Delta\Delta(t)$) (B). Ratio of TM:TE (c). Histamine and dopamine produce a greater shift in the TM mode, consistent with vertical alignment or partial insertion into the membrane. Glycine and GABA show minimal polarization-dependent differences, suggesting limited interfacial engagement.

On/off rates for membrane binding NTs

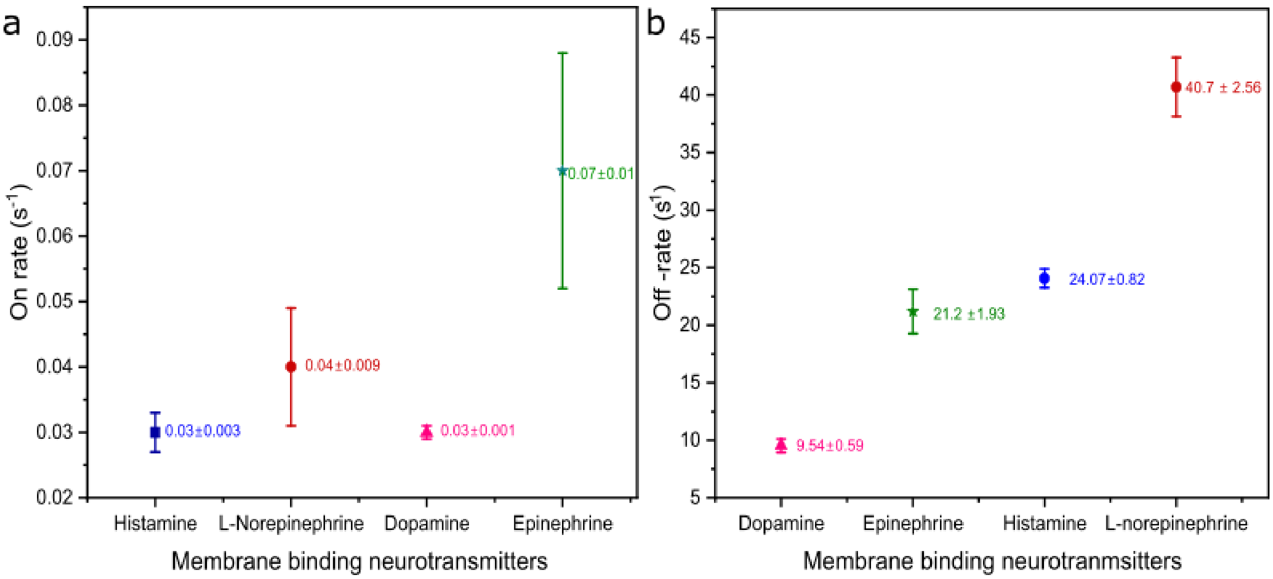

Figure 11: **Spike on-rates and off-rates for membrane binding neurotransmitters** **a**, Spike on-rates determined from survivor function plots for membrane-binding neurotransmitters. **b**, Spike off-rates extracted from dwell time survivor functions. All measurements were performed at a neurotransmitter concentration of 100 fM.

Fitting Parameters

Effect Size Analysis of On-Rates

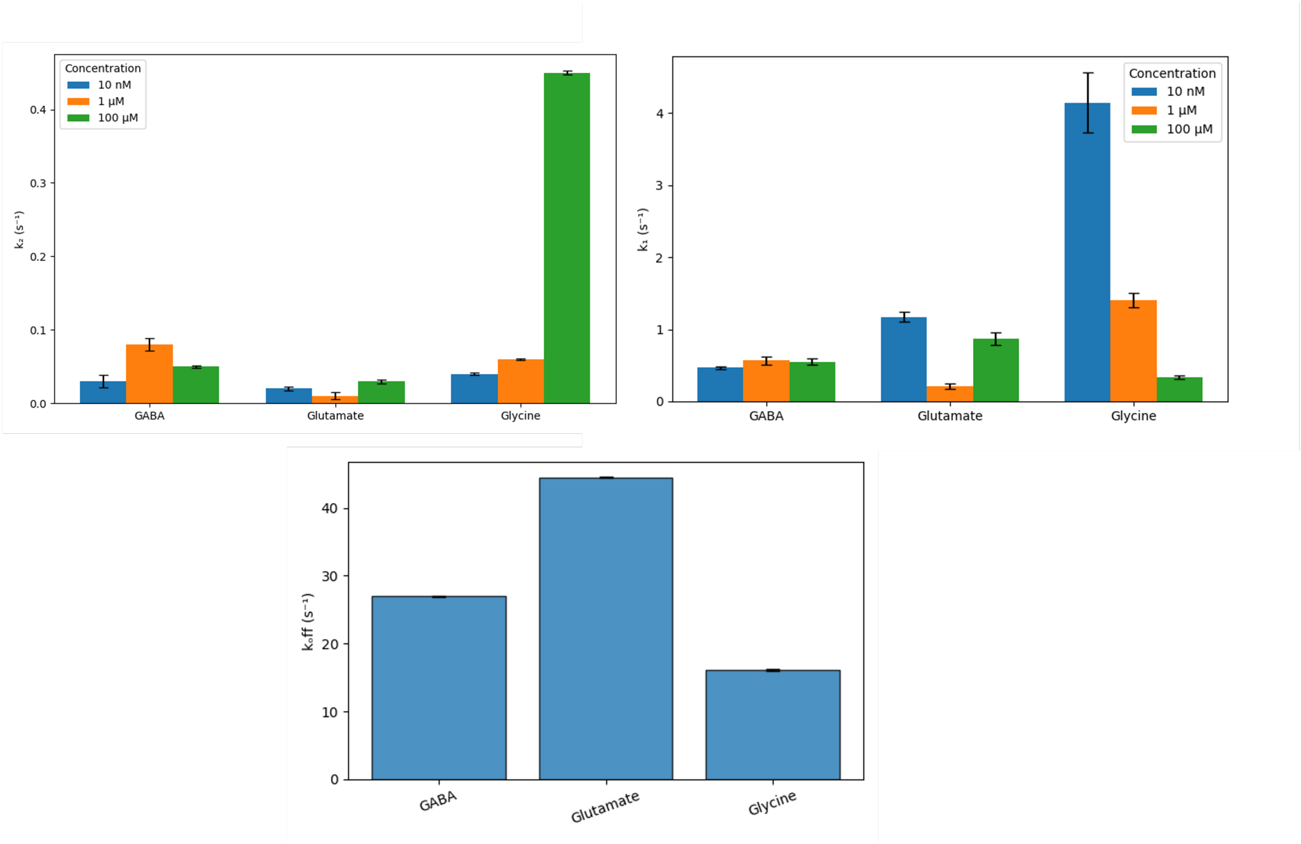

Figure 12: Summary of kinetic parameters for neurotransmitter–membrane interactions derived from single-molecule WGM sensing. (Top) k_2 on-rates for GABA, glutamate, and glycine at 10 nM, 1 μM, and 100 μM. k_1 on-rates for the same neurotransmitters across concentrations, reflecting rapid association events. (bottom) Off-rates (k_{off}) at 10 nM, highlighting differences in dissociation kinetics among amino acid neurotransmitters. Error bars represent 95% confidence intervals from fitting uncertainties.

Pairwise effect sizes and 95% confidence intervals (CI) were computed for $k_1 on$ and $k_2 on$ across GABA, glutamate, and glycine at concentrations. The results are shown in Figure 12 and summarised below:

- **10 nM:**
  - k_1: All pairwise comparisons were significant (no CI overlap).
  - k_2: GABA vs Glutamate and GABA vs Glycine showed CI overlap (not significant); Glutamate vs Glycine was significant.
- **1 μM:**

- k_1: All pairwise comparisons were significant.
- k_2: All comparisons were significant, although GABA vs Glycine had a small effect size (CI [0.004, 0.036] s^{-1}).

- **100 μM:**
  - k_1: All pairwise comparisons were significant.
  - k_2: All pairwise comparisons were significant.

These results confirm that differences in k_1 are consistent across concentrations, while k_2 differences emerge more clearly at higher concentrations.

Fitting parameters summary

The tables below show the fitting parameters for the exponential fits performed in the survivor analyses.

Table 1: On-rate fit parameters (double exponential) for membrane non-binding neurotransmitters

Neurotransmitter	Concentration	A	B	k_1 (s^{-1})	k_2 (s^{-1})	Reduced χ^2
GABA	10 nM	0.77 ± 0.02	0.19 ± 0.02	2.10 ± 0.09	29.5 ± 7.3	6.03×10^{-4}
	1 μM	0.53 ± 0.03	0.45 ± 0.03	1.74 ± 0.14	11.2 ± 1.0	4.54×10^{-4}
	100 μM	0.31 ± 0.01	0.67 ± 0.01	1.82 ± 0.15	17.5 ± 0.51	2.74×10^{-4}
Glutamate	10 nM	0.67 ± 0.01	0.34 ± 0.01	0.77 ± 0.05	21.7 ± 3.0	6.98×10^{-4}
	1 μM	0.46 ± 0.07	0.29 ± 0.06	4.69 ± 0.76	75.75 ± 27.2	7.01×10^{-3}
	100 μM	0.48 ± 0.02	0.46 ± 0.02	1.10 ± 0.12	29.8 ± 2.4	1.34×10^{-3}
Glycine	10 nM	0.62 ± 0.05	0.62 ± 0.008	0.24 ± 0.02	24.6 ± 0.83	7.10×10^{-4}
	1 μM	0.34 ± 0.01	0.64 ± 0.008	0.68 ± 0.04	15.5 ± 0.33	2.44×10^{-4}
	100 μM	0.43 ± 0.02	0.50 ± 0.02	2.90 ± 0.20	21.1 ± 1.1	4.38×10^{-4}

Table 2: Off-rate fit parameters (single exponential) for membrane non-binding neurotransmitters at 10 nM

Neurotransmitter	Concentration	A	k (s)	Reduced χ^2	R-Square
GABA	10 nM	2.65 ± 0.07	0.0370 ± 0.0057	9.97×10^{-4}	0.989
Glutamate	10 nM	2.06 ± 0.04	0.0225 ± 0.0045	7.96×10^{-4}	0.990
Glycine	10 nM	1.96 ± 0.03	0.0622 ± 0.0099	7.57×10^{-4}	0.991

Table 3: On-rate fit parameters (single exponential) for membrane-binding neurotransmitters.

Neurotransmitter	A	k (s)	Reduced χ^2	R-Square
Histamine	0.85 ± 0.02	26.57 ± 2.16	4.63×10^{-3}	0.950
Dopamine	0.89 ± 0.02	38.97 ± 2.55	2.69×10^{-3}	0.972
Epinephrine	0.79 ± 0.04	12.64 ± 2.96	1.45×10^{-2}	0.853
L-Norepinephrine	0.85 ± 0.05	22.17 ± 4.63	9.79×10^{-3}	0.909

Table 4: Off-rate fit parameters (single exponential) for membrane-binding neurotransmitters.

**Neurotransmitter**	**A**	**k (s)**	**Reduced χ^2**	**R-Square**
Histamine	2.15 ± 0.08	0.0415 ± 0.0014	1.35×10^{-3}	0.985
Dopamine	1.43 ± 0.07	0.1048 ± 0.0065	3.25×10^{-3}	0.966
Epinephrine	2.39 ± 0.26	0.0470 ± 0.0043	3.93×10^{-3}	0.960
L-Norepinephrine	3.34 ± 0.32	0.0246 ± 0.0016	1.97×10^{-3}	0.981

Eyring Analysis of Kinetic Data

Temperature-dependent rate constants $k(T)$ were analyzed using the Eyring transition state model to extract activation enthalpy ($\Delta H^{\ddagger}$), entropy ($\Delta S^{\ddagger}$), and Gibbs free energy barriers ($\Delta G^{\ddagger}$) for GABA interactions with EggPC membranes. The Eyring equation is given by

$$k(T) = \frac{k_B T}{h} \exp\left(\frac{\Delta S^{\ddagger}}{R}\right) \exp\left(-\frac{\Delta H^{\ddagger}}{RT}\right), \tag{4}$$

where k_B is the Boltzmann constant, h is Planck's constant, R is the universal gas constant, and T is the absolute temperature.

Taking the natural logarithm of $k(T)/T$ yields a linear form

$$\ln\left(\frac{k}{T}\right) = \ln\left(\frac{k_B}{h}\right) + \frac{\Delta S^{\ddagger}}{R} - \frac{\Delta H^{\ddagger}}{R}\frac{1}{T}. \tag{5}$$

Linear fits of $\ln(k/T)$ versus $1/T$ provide the slope $-\Delta H^{\ddagger}/R$ and intercept $\ln(k_B/h) + \Delta S^{\ddagger}/R$. Gibbs free energy barriers were then calculated at $T = 298$ K via

$$\Delta G^{\ddagger} = \Delta H^{\ddagger} - T\Delta S^{\ddagger}. \tag{6}$$

Uncertainties in $\Delta H^{\ddagger}$ and $\Delta S^{\ddagger}$ were obtained from the covariance matrix of the linear regression, and the uncertainty in $\Delta G^{\ddagger}$ was propagated as

$$\sigma_{\Delta G^{\ddagger}} = \sqrt{\sigma^2_{\Delta H^{\ddagger}} + (T\,\sigma_{\Delta S^{\ddagger}})^2}. \tag{7}$$

The resulting activation parameters are summarized in Table 5. Due to the limited temperature range and measurement precision, the uncertainties are large and the parameters should be interpreted qualitatively rather than quantitatively.

Estimation of Neurotransmitter Surface Density and Binding Ratio

To estimate the local neurotransmitter (NT) concentration at the sensor surface, we assumed that all NT binding events occurred exclusively at the tips of plasmonic nanorods (NRs), where the Whispering Gallery Mode (WGM) field is most strongly enhanced. This is an oversimplification since binding does not occur at the nanorod surface but instead in or

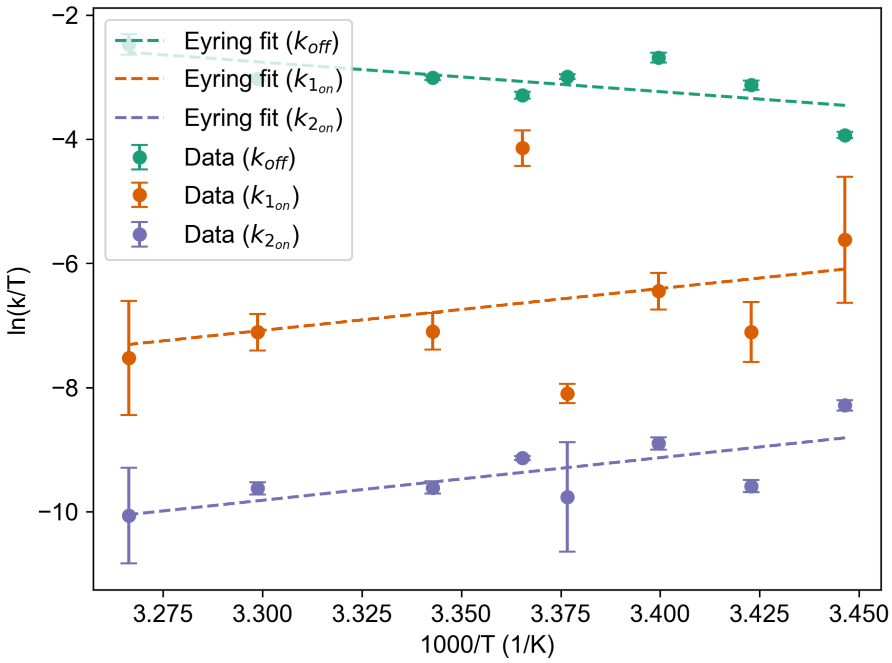

Figure 13: Eyring plots for k_{off}, k_{1on}, and k_{2on}. Data points show $\ln(k/T)$ versus $1000/T$ with standard error bars. Dashed lines denote linear fits used to extract $\Delta H^\ddagger$ and $\Delta S^\ddagger$.

Table 5: Activation parameters from Eyring analysis of GABA interactions with EggPC membranes. Values are reported as mean ± standard error (SE).

Series	$\Delta H^\ddagger$ (kJ mol^{-1})	$\Delta S^\ddagger$ (J mol^{-1} K^{-1})	$\Delta G^\ddagger$ (298 K) (kJ mol^{-1})
k_{off}	36.3 ± 29.5	97.6 ± 99.2	7.2 ± 42.0
k_{1on}	-34.1 ± 68.0	-170.1 ± 228.9	16.6 ± 96.2
k_{2on}	-47.6 ± 23.3	-238.4 ± 78.4	23.4 ± 33.0

around the lipid bilayer. However this can serve as a relevant estimate of the upper limit of NT binding.

Each nanorod is taken to be 10 nm in diameter and 35 nm in length, with both ends approximated as hemispherical tips. NT binding is presumed to occur only at these hemispherical tips.

Nanorod Tip Surface Area. The surface area of a hemisphere is given by:

$$A_{\text{tip}} = 2\pi r^2$$

For a radius of $r = 5$ nm:

$$A_{\text{tip}} = 2\pi(5\text{ nm})^2 = 157.08\text{ nm}^2$$

Each nanorod has two hemispherical tips, giving:

$$A_{\text{NR}} = 2 \cdot 157.08 = 314.16\text{ nm}^2$$

For 4 nanorods:

$$A_{\text{total}} = 4 \cdot 314.16 = 1256.64 \text{ nm}^2$$

Neurotransmitter Surface Density. Assuming 56 NT binding events were detected:

$$\rho_{\text{NT}} = \frac{56 \text{ NTs}}{1256.64 \text{ nm}^2} \approx 0.0446 \text{ NTs/nm}^2$$

Converted to per square centimeter:

$$\rho_{\text{NT}} = 0.0446 \text{ NTs/nm}^2 \times 10^{14} \text{ nm}^2/\text{cm}^2 = 4.46 \times 10^{12} \text{ NTs/cm}^2$$

Estimated Binding Ratio (NTs per Lipid). Assuming a lipid packing density of 3.5×10^{14} lipids/cm^2[3], the NT-to-lipid ratio is:

$$\frac{4.46 \times 10^{12} \text{ NTs/cm}^2}{3.5 \times 10^{14} \text{ lipids/cm}^2} \approx 0.0127 \text{ NTs per lipid}$$

This corresponds to approximately 1 NT for every 79 lipids in the detection region.

References

[1] Aneeth Kakkanattu Arunkumar, Jolly Xavier, and Frank Vollmer. Opto- plasmonic whispering gallery mode (wgm) sensors for the detection of gaba. In Frontiers in Optics + Laser Science 2023 (FiO, LS), page JTu5A.1. Optica Publishing Group, 2023. doi: 10.1364/FIO.2023.JTu5A.1. URL https://opg.optica.org/abstract.cfm?URI=FiO-2023-JTu5A.1.

[2] Aneeth Kakkanattu Arunkumar, Ekaterina Zossimova, Michael Walter, Srikanth Pedireddy, Jolly Xavier, and Frank Vollmer. Probing the single neurotransmitters with the wgm microcavity-hybridized plasmonic nanospiked antennas, 2025. URL https://arxiv.org/abs/2507.10146.

[3] John F. Nagle, Stephanie Tristram-Nagle. Structure of lipid bilayers. Biochim. Biophys. Acta 1469, 159–195 (2000).

Acknowledgements

This research was supported by the Biotechnology and Biological Sciences Research Council (BBSRC) under the BBSRC-NSF/BIO program (grant number BB/V004166/1) and by EPSRC (grant number EP/T002875/1). A.K.A. acknowledges the support provided by the Research Training Support Grant (RTSG) from the University of Exeter.

Author contributions statement

T.L.D. and A.K.A. designed and performed the experiments and analysed the data. R.C. and E.S. conducted WGM measurements. All authors contributed to writing and editing the manuscript.

Additional information

The authors declare no competing interests.
